# A fibrolytic potential in the human ileum mucosal microbiota revealed by functional metagenomic

**DOI:** 10.1038/srep40248

**Published:** 2017-01-16

**Authors:** Orlane Patrascu, Fabienne Béguet-Crespel, Ludovica Marinelli, Emmanuelle Le Chatelier, Anne-Laure Abraham, Marion Leclerc, Christophe Klopp, Nicolas Terrapon, Bernard Henrissat, Hervé M. Blottière, Joël Doré, Christel Béra-Maillet

**Affiliations:** 1 Micalis Institute, INRA, AgroParisTech, Université Paris-Saclay, 78350 Jouy-en-Josas, France; 2 Metagenopolis, INRA, 78350 Jouy-en-Josas, France; 3 Plate-forme bio-informatique Genotoul, Mathématiques et Informatique Appliquées de Toulouse, INRA, Castanet-Tolosan, France; 4 CNRS UMR 7257, Université Aix-Marseille, 13288 Marseille, France; 5 INRA, USC 1408 AFMB, 13288 Marseille, France; 6 Department of Biological Sciences, King Abdulaziz University, Jeddah, Saudi Arabia

## Abstract

The digestion of dietary fibers is a major function of the human intestinal microbiota. So far this function has been attributed to the microorganisms inhabiting the colon, and many studies have focused on this distal part of the gastrointestinal tract using easily accessible fecal material. However, microbial fermentations, supported by the presence of short-chain fatty acids, are suspected to occur in the upper small intestine, particularly in the ileum. Using a fosmid library from the human ileal mucosa, we screened 20,000 clones for their activities against carboxymethylcellulose and xylans chosen as models of the major plant cell wall (PCW) polysaccharides from dietary fibres. Eleven positive clones revealed a broad range of CAZyme encoding genes from *Bacteroides* and Clostridiales species, as well as Polysaccharide Utilization Loci (PULs). The functional glycoside hydrolase genes were identified, and oligosaccharide break-down products examined from different polysaccharides including mixed-linkage β-glucans. CAZymes and PULs were also examined for their prevalence in human gut microbiome. Several clusters of genes of low prevalence in fecal microbiome suggested they belong to unidentified strains rather specifically established upstream the colon, in the ileum. Thus, the ileal mucosa-associated microbiota encompasses the enzymatic potential for PCW polysaccharide degradation in the small intestine.

The human intestine is a long and segmented part of the gastrointestinal (GI) tract, characterized by the small intestine in the proximal part, and the large intestine (colon) in the distal part. Both are colonised by a complex and diversified microbial community, the intestinal microbiota, accounting for 10^2^ to 10^8^ bacteria/g in the small intestine depending on duodenum, jejunum or ileum segments^1^ and 10^11^ bacteria/g of content in the colon^2^. Biodiversity and functions of the intestinal microbiota have been studied for many years, with considerable progress during the last decade, with the emergence of culture-independent ‘omics’ approaches^3^. The intestinal microbiota is now considered as an “organ” with structural alterations regarded as a causal driver or consequence in several diseases^4^.

So far, most studies have focused on the fecal microbiota for obvious practical aspects of stool sample collection. Hundreds of different microbial species have been described using cultural and non-cultural approaches^5^. Close to ten million microbial genes have been reconstructed from metagenomics data collected from 1,267 human fecal samples^6^, a number that outcompetes the human gene catalogue by 400-fold^7^, and offers numerous additional pathways that the host does not encode. This complex community is characterized by Firmicutes and Bacteroidetes, as the two main phyla, together with less representative ones like *Actinobacteria, Verrucomicrobia* and *Proteobacteria*^8^. Microbes are organized in a trophic network, playing a key role in nutrition and health by providing nutrients and energy to the host through anaerobic fermentations of dietary components^2^,^9^.

A recent publication emphasized the importance to also expand microbiota studies to the small intestine, despite limited accessibility of this site^10^. Starting from biopsies of mucosal tissues or luminal effluent from ileostomized subjects, several studies focused on the terminal part of the small intestine, and considered the distal ileum as an interesting site for digestion^1^,^11^,^12^,^13^,^14^,^15^,^16^,^17^. Indeed, the ileum harbors a dense bacterial community (up to 10^8^ bacteria/g) composed of Bacteroidetes (*Bacteroides, Prevotella*), *Proteobacteria* and *Actinobacteria* as well as Firmicutes (*Lachnospiraceae, Bacillus, Streptococcus, Faecalibacterium*)^1^,^11^,^12^,^14^. This large bacterial community takes advantage of the favorable physiochemical environment: pH 6.5/7.0, redox potential −200 mV, 37 °C, reduced motility and increased stasis^18^. Furthermore, the mucus layer covering the epithelial cells is very different between the small and large intestine, it is less dense, thinner, with different glycosylation patterns i.e. less fucosylated and more sialylated and sulfated^19^,^20^. Because of their close localization with respect to host cells and the stability of mucosal dominant bacterial species^21^, the microbiota of the ileal mucosa is likely to play a role in the regulation of intestinal homeostasis and immune responses^22^. Nevertheless, the ecological role of these microorganisms and their relationships with the host still need to be clarified. Zoetendal *et al*.^1^ revealed overrepresented genes related to fermentation pathways and rapid uptake of simple sugars in ileal effluent of ileostomized individuals. Furthermore, bacteria associated to the ileal mucosa are able to degrade prebiotic-oligosaccharides with less than 10 monosaccharide units such as fructo-, galacto-, xylo-oligosaccharides and lactulose^23^. In contrast to the colonic microbiota, the potential of the ileal microbiota for dietary fibre degradation, particularly complex glycan, has not been explored yet.

Most of the complex glycans reaching our small intestine are plant cell wall (PCW) polysaccharides from fruits and vegetables. They are considered dietary fibres as the human genome does not encode any enzyme for their breakdown to simple sugars^24^,^25^. Yet their microbial degradation is essential for the release of fermentation products like organic acids, gases and short-chain fatty acids (SCFAs) and hence to provide benefits for the host^26^. Dietary fibres are metabolized by the intestinal bacteria using a large number of degradative enzymes such as glycoside hydrolases, polysaccharide lyases and carbohydrate esterases collectively termed carbohydrate-active enzymes (CAZymes) and listed in the CAZy database (http://www.cazy.org). CAZymes are classified in different families based on amino acid sequence similarities, protein folds and hydrolytic mechanisms^27^. In Bacteroidetes, CAZymes that are necessary to the digestion of a particular glycan structure are often part of Polysaccharide Utilization Loci (PULs), characterized by genes encoding the outer membrane SusCD-like proteins involved in polysaccharide binding and oligosaccharide import^28^,^29^,^30^.

Recent *in-silico* meta-studies indicate that most of the documented CAZyme families are represented in hundreds of human gut microbiomes, making this ecosystem one of the major known sources of CAZymes^25^,^31^. Moreover, most of detected CAZymes are only predicted enzymes and an experimental validation of their function is needed for a better understanding of the wide degradation pathways^32^. To further extend our understanding of the role of the small intestine microbiota in complex glycan catabolism, we explored the capacity of the mucosa-associated microbiota of the ileum to break down complex and diversified carbohydrates by synthesizing the appropriate enzymes. Using a functional screening against polysaccharides representatives of the major complex PCW components (xylans, mixed-linkage β-glucans and a cellulose derivative), we established a functional repertoire of CAZymes and PULs from the ileal mucosa-associated microbiota. We suggest that microorganisms from this intestinal site may also participate to the degradation of microbiota-accessible carbohydrates (MACs) in the small intestine, and especially complex glycans from dietary fibres.

## Results

### Functional screening of a mucosal metagenomic library from human ileum reveals a broad range of CAZymes

**Figure 1:**
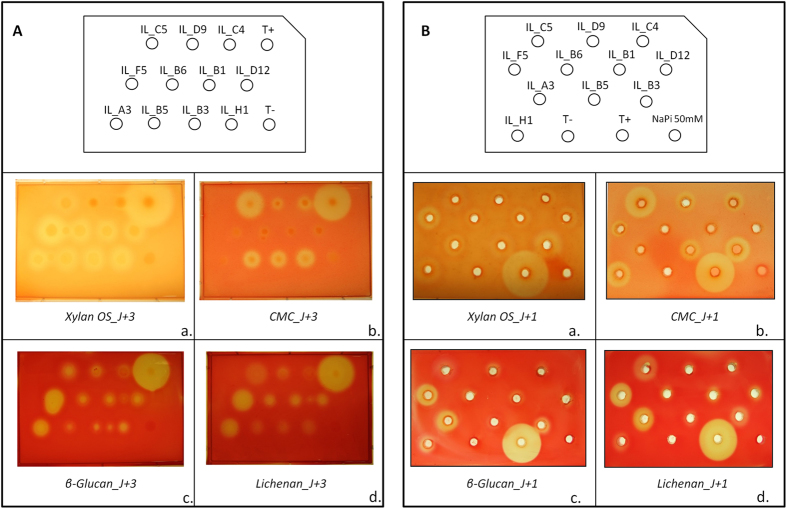
Detection of xylanase (a), carboxymethylcellulase (b), β-glucanase (c) and lichenase (d) activities of eleven metagenomic clones from the human ileal mucosa library, using colony screenings (**A**) and agar-well plate assays with concentrated bacterial extracts (**B**). Agar plate LB medium was supplemented with 0.5% (w/v) oat spelt xylans, carboxymethylcellulose (CMC), β-glucan or lichenan. Plates were incubated 1 (for B) to 3 days (for A) at 37 °C. Clear halo around a colony or well indicates a positive colony or sample for the corresponding glycoside hydrolase activity. Negative (T−) and positive (T+) controls for GH activities were added in each plate.

A metagenomic library produced from the ileal mucosa-associated microbiota was used for this study and comprised *E. coli* recombinant fosmid clones with about 40 kb DNA inserts^33^. In order to explore the fibrolytic systems of these microorganisms, 20,000 metagenomic clones were screened for their PCW polysaccharides degrading capacity. Eleven positive metagenomic clones with significant and reproducible enzymatic activities were detected by the presence of clear halos around the colonies. Five clones (IL_F5, IL_B6, IL_B1, IL_D12 and IL_A3) displayed only xylanase activity and six clones (IL_C5, IL_D9, IL_C4, IL_B5, IL_B3 and IL_H1) produced both endoglucanase and xylanase activities (Fig. 1A). Xylanase activity of IL_D9 and IL_C4 was moderate. In a second targeted screening, all the eleven positive clones were shown to degrade mixed-linkage polysaccharides like β-1,3-1,4-glucan and lichenan (Fig. 1A) whereas none of them displayed xyloglucanase activity.

The activities of these clones were validated using bacterial and extracellular concentrated proteins incubated with the different substrates using supplemented agar-well plating (Fig. 1B). Activities were always associated to bacterial cells rather than extracellular fractions (not shown). A weak carboxymethylcellulase activity was observed for the IL_F5, IL_B6, IL_B1, IL_D12 and IL_A3 concentrated bacterial samples, but corresponded to basal endoglucanase activity of the *E. coli* host strain.

**Figure 2:**
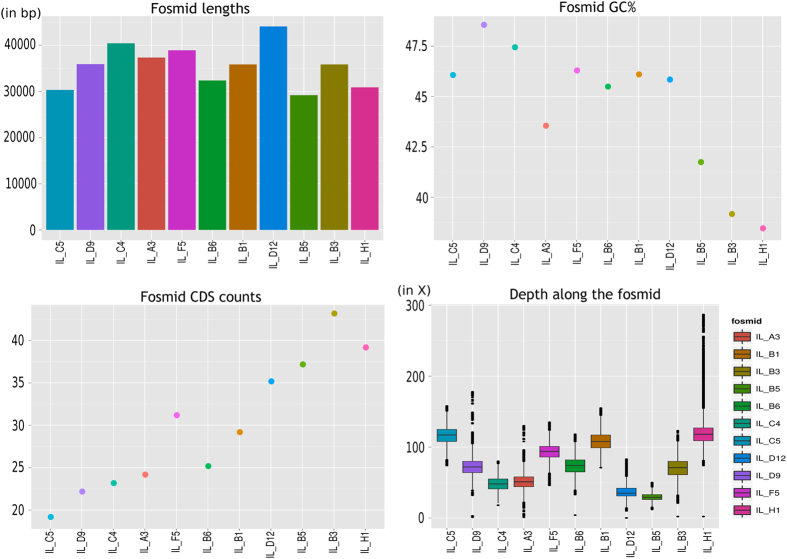
Statistics for the eleven positive clones including length, GC% of the metagenomic inserts, number of CDS per clone and nucleotide coverage (read projection on assemblied metagenomic insert sequences).

The inserts from the eleven positive clones were sequenced and meticulous assembly led to unique contigs of between 29 and 44 kb depending on the metagenomic insert, with a GC% comprised between 38 and 48 (Fig. 2). Contig coverage ranging from 29 to 118X allowed accurate assemblies with a predicted coding DNA sequence (CDS) number ranging from 19 to 43 depending on the clone (Fig. 2).

**Figure 3:**
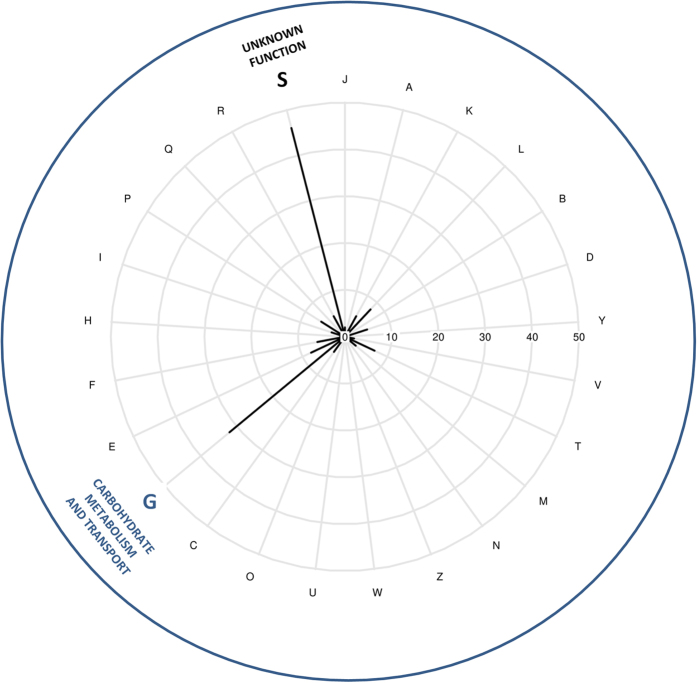
Radial plot of EggNOG functional category assignments of all the non-redundant CDS from positive metagenomic clones. The proteins not assign to any EggNOG are not shown in this figure. INFORMATION STORAGE AND PROCESSING: [J] Translation, ribosomal structure and biogenesis; [A] RNA processing and modification; [K] Transcription; [L] Replication, recombination and repair; [B] Chromatin structure and dynamics. CELLULAR PROCESSES AND SIGNALING: [D] Cell cycle control, cell division, chromosome partitioning; [Y] Nuclear structure; [V] Defense mechanisms; [T] Signal transduction mechanisms; [M] Cell wall/membrane/envelope biogenesis; [N] Cell motility; [Z] Cytoskeleton; [W] Extracellular structures; [U] Intracellular trafficking, secretion, and vesicular transport; [O] Posttranslational modification, protein turnover, chaperones. METABOLISM: [C] Energy production and conversion; [G] Carbohydrate transport and metabolism; [E] Amino acid transport and metabolism; [F] Nucleotide transport and metabolism; [H] Coenzyme transport and metabolism; [I] Lipid transport and metabolism; [P] Inorganic ion transport and metabolism; [Q] Secondary metabolites biosynthesis, transport and catabolism. POORLY CHARACTERIZED: [R] General function prediction only; [S] Function unknown.

Predicted proteins of the eleven clones were assigned to 18 Non-supervised Orthologous Groups, named eggNOG categories (Fig. 3 and Table S1). The carbohydrate transport and metabolism [G] category is a major assignment in the radial plot. Each clone follows individually the same trend, except the clone IL_B5 (Fig. S1). Many proteins were assigned to the unknown function [S] category, suggesting a lack of knowledge and reliable annotations regarding these proteins, or/and missing representative categorized proteins in the eggNOG database.

**Figure 4:**
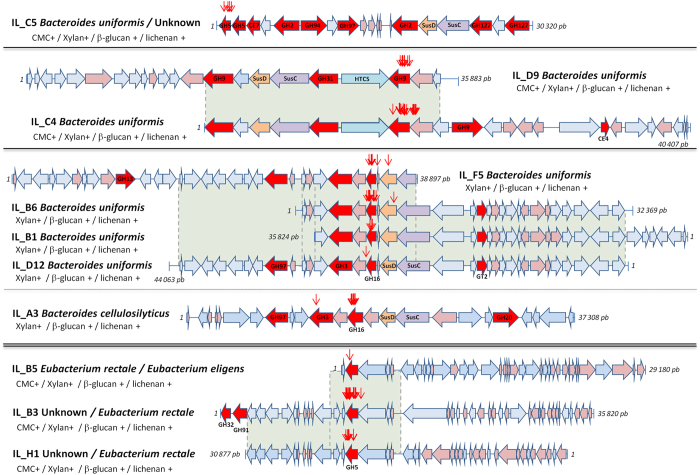
Genetic maps of the eleven positive metagenomic clones. Clones with an overlapping sequence (highlighted in green) are grouped and delimited by dark horizontal lines. Length of each clone is indicated with the first and last nucleotides. Each CDS is numbered according to its position on the clone. Annotated CDS for CAZymes (GH, CE, GT) are illustrated in red, SusCD-like proteins in orange, hypothetical proteins in pink. Transposons leading to an inactivated GH phenotype of the metagenomic clones are shown with red arrows.

To evaluate the encoding enzymatic equipment of the positive clones, genetic maps of the eleven metagenomic inserts were built (Fig. 4). Taxonomic assignments of nucleic and protein sequences using Blast facilities suggested that eight metagenomic inserts originate from the *Bacteroides* genus (Table S2). At the species level, IL_D9/C4. and IL_F5/B6/B1/D12 are clearly related to human intestinal *Bacteroides uniformis* whereas IL_C5 is affiliated to *B. uniformis* for half of the sequence only. Clone IL_A3 is phylogenetically close to the cellulolytic human gut bacterium *Bacteroides cellulosilyticus*.

The three remaining fosmids, IL_B5, IL_B3 and IL_H1 are related to Gram positive Firmicutes species belonging to the Clostridiales, in the former low GC% *Clostridium* cluster XIVa of Collins *et al*.^34^ (Table S2). This is consistent with the low GC% of the three metagenomic sequences (Fig. 2). These clones are not homologous of known unique genomes. In addition, 20 to 60% of their CDS cannot be assigned at the species-level (Table S2). IL_B5 is related to *Eubacterium rectale* for first third of its sequence, and then to *Eubacterium eligens*. IL_B3 and IL_H1 are mainly related to *E. rectale* although part of the inserts has no clear affiliation.

Finally, 43 CDS were not affiliated at the species level, according to stringent similarity threshold at the nucleotide level (>97%). This suggests that the inserts originated from yet undescribed strains. In the same way, the CDS annotation was not conclusive, mainly for the Clostridiales hit clones, and evidenced numerous hypothetical proteins (HP).

**Figure 5:**
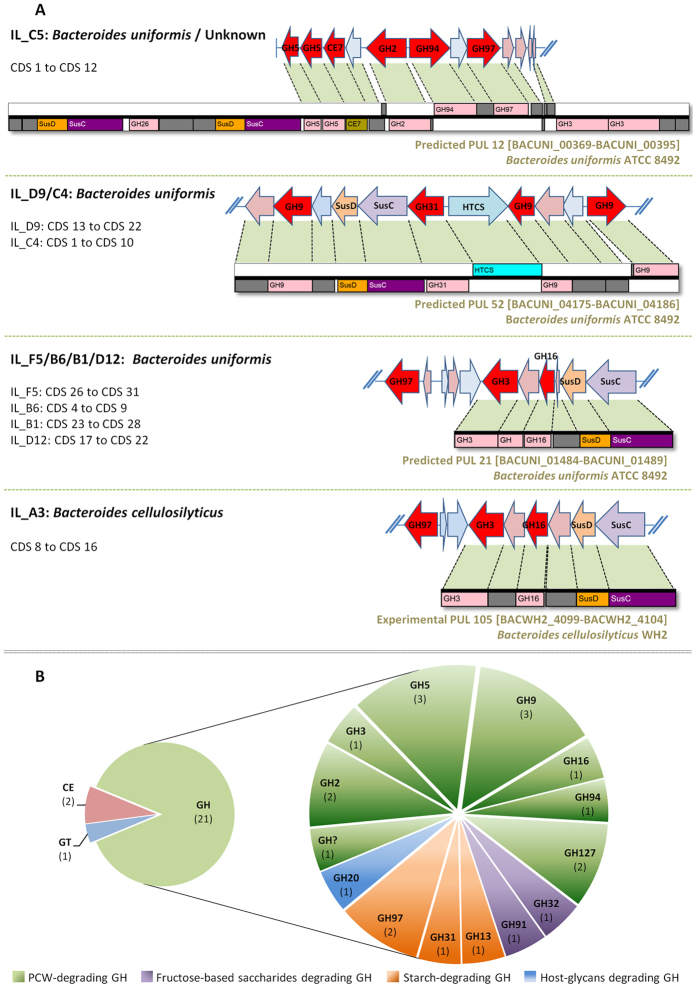
PUL organization in the *Bacteroides* clones (**A**) and CAZyme repartition in the eleven positive metagenomic clones (**B**).

Nevertheless, the CAZyme annotation allowed the identification of putative GH, CE and GT enzymes in each clone, and the accurate validation of their family classification (Tables 1 and S2). The eight clones related to *Bacteroides* species were examined first for their CAZymes and PUL systems. The metagenomic insert IL_C5 comprises the highest number of putative GH with eight enzymes from five different families (GH2, GH5_2 and GH5_7, GH94, GH97, GH127), and one CE (CE7) (Fig. 4 and Table S2). Regarding the predicted function of its GH, IL_C5 seemed to bear the capacity to disrupt several types of carbohydrates containing glucose, xylose, arabinose, mannose and galactose (Table S3). The IL_C5 CDS 1 to 10 encoded proteins present high similarity with a part of the predicted PUL 12 (BACUNI_00369-BACUNI_00395) of *B. uniformis* ATCC 8492 with unknown function by sharing six encoded GH and CE (Fig. 5A and Table S2), whereas the second part of IL_C5 with no clear taxonomic affiliation is not included in a PUL.

The IL_D9 and IL_C4 clones have an overlapping metagenomic sequence of 19.6 kb, sharing SusCD-like proteins and three putative GH related to GH9 and GH31 (Fig. 4). This region is homologous to the predicted PUL 52 (BACUNI_04175-BACUNI_04186) of *B. uniformis* ATCC 8492 (Fig. 5A and Table S2) with unknown function, and includes a member of the hybrid two-component system (HTCS) for carbohydrate sensing and signal transduction. IL_C4 bears additional GH9 and CE4 sequences which could be involved in cellulose degradation and xylan deacetylation, respectively. Among the three GH9 enzymes, a family which mainly consists of endoglucanases, two sequences are more remotely related to the classical GH9 profile with only 22% amino acid (aa) identity with endoglucanases from *Ruminiclostridium thermocellum* (accession number CAA28255.1) and *Clostridium cellulolyticum* (ACL75133.1) (Table 1).

Clones IL_F5, IL_B6, IL_B1 and IL_D12 also shared a limited region (around 9.6 kb) of their metagenomic insert, comprising a GH3 sequence, a member of an unknown family of GHs, a GH16 and SusCD-like proteins (Fig. 4), similar to the predicted PUL 21 (BACUNI_01484- BACUNI_01489) of *B. uniformis* ATCC 8492 (Fig. 5A and Table S2).

IL_A3 from *B. cellulosilyticus* contains three putative GH from families 3, 16, and 97 targeting PCW polysaccharides, and a GH20 more probably dedicated to the glycosaminoglycan metabolism (Fig. 4). The SusCD-like proteins associated to the GH3 and GH16 are part of the experimentally validated PUL 105 (BACWH2_4099-BACWH2_4104)^35^ of *B. cellulosilyticus* WH2 (Fig. 5A and Table S2). The CDS encoding an unknown protein located between the GH3 and the GH16 has no similarity with the corresponding putative GH of the IL_F5/B6/B1/D12 clones.

The three Firmicutes clones bear a GH5_2 gene, a subfamily that essentially contains endoglucanases. The GH5_2 protein is identical in IL_B3 and IL_H1, and shares 98% aa identity with the IL_B5 GH5. IL_B3 contains two additional enzymes from families GH32 and GH91 most likely implicated in the metabolism of inulin, a natural storage polymer of fructose.

Overall, the eleven metagenomic clones reveal a broad range of distinct CAZymes (Fig. 5B). The clones harbors 21 different GHs from thirteen different families, one protein with weak similarity to GH-A clan (restricted to the region containing the catalytic residues and insufficient to be classified in an existing CAZy family), two CE (CE4 and CE7) and the GT2, for a total of 25 enzymes dedicated to carbohydrate metabolism. All GH families are associated to dietary fibre metabolism (Table S3), except the GH20 most likely involved in the degradation of endogenous mucus, and the unclassified GH. The GHs from families 2, 3, 5, 9, 16, 94 and 127 are directly involved in complex PCW polysaccharide breakdown; the GH13, GH31 and GH97 in disruption of starch and starch-derivatives; and the GH32 and GH91 in degradation of inulin.

Whilst all of these enzymes have sequence homologs in the publicly available metagenomic databases, their actual enzymatic properties and substrate specificities remain uncharacterized.

### Xylanase and mixed-linkage glucan activities of the positive clones

Using random transposon mutagenesis of the metagenomic positive clones and secondary enzymatic screenings, GH genes bearing xylanase, carboxymethylcellulase, β-glucanase and lichenase activities were investigated.

#### GHs with xylanase activity

Xylanase activity (red arrows on Fig. 4) was carried by the expected GH5 gene for *Bacteroides* IL_C5 and the three Firmicutes clones IL_B5, IL_B3 and IL_H1. However, mutations in genes encoding for GH16 (for IL_F5/B6/B1/D12) and GH9 (for IL_C4 and IL_B3) also inactivated the xylanase activity. This was surprising since family 9 and 16 GH enzymes are described as hydrolysing β-glucan (mix-linkage glycan) or xyloglucan polysaccharides.

Because the OS xylan was composed of 75% xylose, 10% arabinose and 15% glucose, the likely presence of xyloglucan or β-glucan polysaccharides could introduce false positives in the xylanase activity detection. However, none of the clone exhibited xyloglucanase activity in agar well-plate assays (not shown).

**Figure 6:**
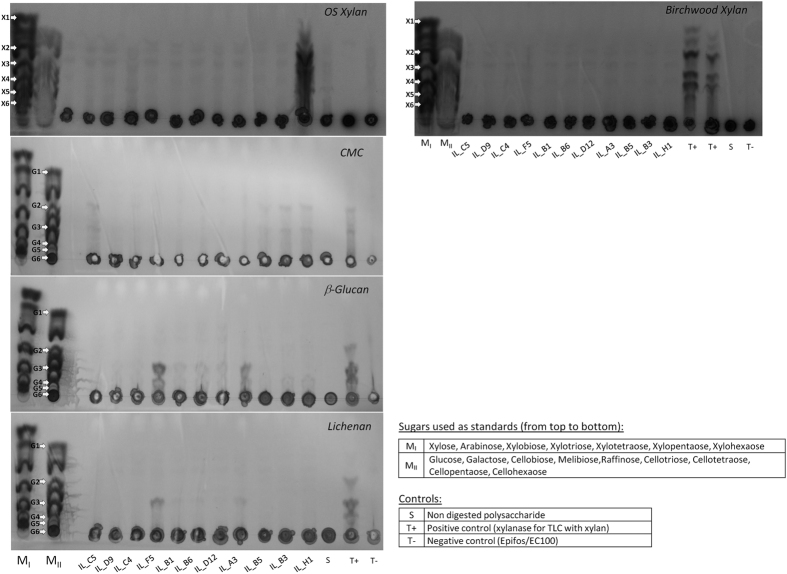
TLC sugar analysis of bacterial extracts from the eleven clones and controls incubated with CMC, mixed-linkage β-glucans or xylans. Standard sugar mixes MI and MII include Xylose (X1), Arabinose, Xylobiose (X2), Xylotriose (X3), Xylotetraose (X4), Xylopentaose (X4) and Xylohexaose (X6) or Glucose (G1), Galactose, Cellobiose (G2), Melibiose, Raffinose, Cellotriose (G3), Cellotetraose (G4), Cellopentaose (G5) and Cellohexaose (G6).

We then demonstrated in TLC with protein extracts that all the positive clones were able to degrade OS xylan and purified birchwood xylan as evidenced by a faint but reproducible simple sugar release (Fig. 6).

#### GHs with (mixed-linkage) endoglucanase activities

Carboxymethylcellulase activity was also supported by genes encoding GH5 and GH9 in six of the eleven clones (Fig. 1): the Bacteroidetes IL_C5 (GH5_2 and GH5_7) and IL_D9/C4 (GH9), and the Firmicutes IL_B5, IL_B3, IL_H1 clones (GH5_2) (Fig. 4, red arrows). Consistently, TLC analysis confirmed a slight production of di- to hexa-saccharides from the CMC degradation, especially by IL_C5 and IL_B5/B3/H1 (Fig. 6).

All the clones were able to degrade β-1,3-1,4-glucan and lichenan with different efficiencies (Fig. 1). The inactivation of genes conferring these activities was always linked to the endoglucanase or xylanase encoding genes, indicating a broader substrate affinity for these enzymes. The strongest β-glucanase and lichenase activities were observed for IL_F5/B6/B1/D12 and IL_A3. Indeed, a clear production of tri- to hexasaccharides from β-glucan and lichenan (with less intensity) was observed for these five clones (Fig. 6). Slight production of oligosaccharides (degree of polymerization > 4) was also observed with β-glucan for the six remaining clones.

### Prevalence and abundance of genes involved in carbohydrate metabolism in human ileal and fecal metagenomes

Firstly, homologs of the 251 non-redundant CDS from the eleven hit clones were searched in the rarely available metagenomic sequences from the human ileum^1^,^23^,^36^.

Homologies (identity percentage > 81%; alignment percentage > 31.7%) were only found with genes from ileal effluent microbiota^1^ for one *Bacteroides* IL_D12 CDS and eight CDS of the Firmicutes IL_B5 (4 CDS), IL_B3 (2 CDS) and IL_H1 (2 CDS) (Table S4). None of these genes bear GH, encoding proteins involved in general metabolism, possibly shared by luminal and mucosa-associated bacteria from the ileum.

Secondly, fifty-two non-redundant CDS encoded proteins from the eleven hit clones comprising CAZymes and other proteins involved in carbohydrate metabolism and transport, were searched in the MetaHit microbial gene catalogue, built from 1,267 fecal metagenomic samples^6^. All the proteins had almost one homologous sequence, except the short IL_F5 CDS 29 protein (Table S5).

**Figure 7:**
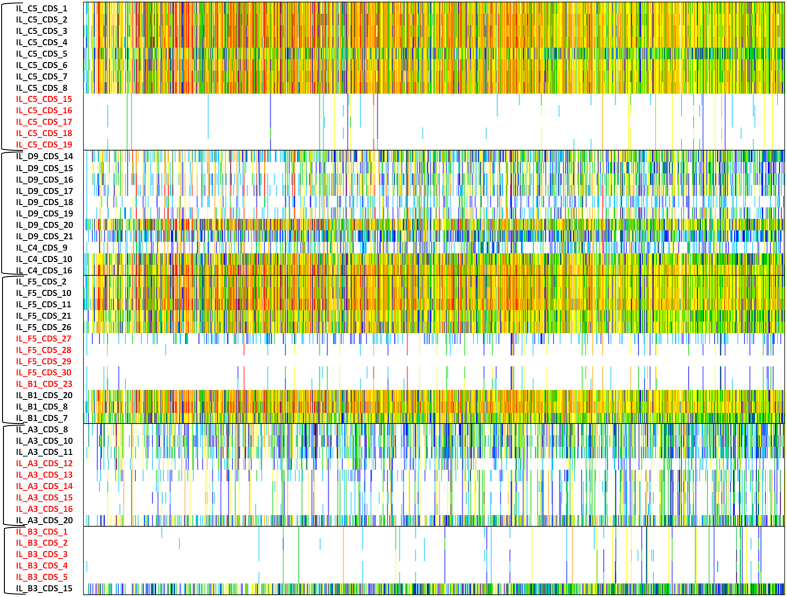
Prevalence and abundance of the metagenomic insert gene homologs found in the MetaHit 9.9 M human gut reference catalogue (including only data from fecal metagenomes because of the lack of ileal sampling). Genes are in rows; individuals, ordered by increasing gene number, are in columns; frequency is indicated by color gradient (white, not detected; light blue to red, increasing abundance with a 4-fold change between colors).

We thus examined the prevalence of these homologs in individuals. Two clusters of genes exhibited an extremely low prevalence in fecal samples, being detected in less than 4% (IL_C5 CDS 15 to 19 and IL_B3 CDS 1 to 5 on heat map; Fig. 7 and Table S5). These genes encode GH and other uncharacterized proteins assigned to carbohydrate metabolism. Another cluster of low prevalence genes (4 to 20%) was evidenced in the overlapping region (IL_F5 CDS 27 to IL_B1 CDS 23) shared by IL_F5, IL_B6, IL_B1 and IL_D12 that is homologous to the predicted PUL 21 (BACUNI_01484- BACUNI_01489) of *B. uniformis* ATCC 8492. A similar cluster of genes with a moderate prevalence (17 to 23%) was evidenced in IL_A3 (CDS 12 to 16), belonging to part of the experimentally validated PUL 105 of *B. cellulosilyticus* WH2.

The taxonomic affiliation of genes with low prevalence was usually different from those of the other genes of the metagenomic inserts. Genes from the same cluster could also be affiliated to different species. All these observations suggest that genes of low prevalence clusters belong to unknown bacterial strains. Finally, the richness in gene content of the individuals had no influence on the gene cluster prevalence, since prevalence was observed with the same proportion in all individuals (Fig. 7).

## Discussion

Using functional metagenomic screening in *E. coli*, our study demonstrated that the human ileal ecosystem harbors an enzymatic machinery able to perform catabolism of complex and diversified PCW polysaccharides from dietary fibres. We thus evidenced a fibrolytic potential in the mucosa-associated bacteria from the ileum.

Our results targeting PCW glycans are complementary to those of previous studies. Until now, the presence of monosaccharides (glucose, galactose or fructose) in the ileum was mainly explained by the digestive action of pancreatic α-amylase and some enterocyte brush-border membrane enzymes against complex carbohydrates^37^. These simple sugars are transported through the epithelial cells of the GI tract to finally reach the portal vein^38^. Using metagenomics, two recent works clearly evidenced, in ileal microbiota, functions related to short oligosaccharide degradation, carbohydrate uptake, and central carbon and energy metabolism^1^,^23^. This indicates that the ileal microbiota is involved in sugar utilization and harbors fermentation pathways leading to energy generation for growth. However, fibrolytic enzymes able to disrupt long and complex PCW polysaccharides were not explored in the ileum. Our study provided the first evidence of this function.

Besides the physicochemical properties of the ileum, the presence of mucus covering the intestinal epithelial cells favors the establishment of mucosa-associated microorganisms which differ from the luminal microbiota^39^. Microorganisms associated with the lumen fluctuate depending on carbohydrate intakes or during the day^1^,^16^. On the contrary, the resident mucosal microbiota is considered to be more stable^40^, counterbalancing a shortened transit time compared to the large bowel, and finally being in contact with various and renewed nutrients. Indeed, 60% of the postprandial ileal contractions are stationary, fulfilling the function of mixing^41^ and spreading the chyme over the mucosal surface while motility is reduced for a longer contact time. Indeed, contractions are less intense and frequent in the ileum than in the colon^42^ also favoring the mixing of nutrients. The ileum mucosa is thus a convenient intestinal niche for the establishment of a fibrolytic community.

The metagenomic sequences encoding fibrolytic enzymes we identified in this study support this statement. Genomes were taxonomically related to *Bacteroides* species and to Clostridiales close to *Eubacterium* species.

*Bacteroides* is a dominant genus within the intestinal microbiota. Several species like *Bacteroides thetaiotaomicron* and *Bacteroides ovatus* harbor a large repertoire of genes for sensing, binding and metabolize carbohydrates^43^,^44^, thus developing efficient strategies to harvest glycans. Our study emphasized a fibrolytic potential in *B. uniformis* and closely related yet undescribed strains. Indeed, among the eleven positive clones, seven were affiliated to this species suggesting that *B. uniformis* could play a role in PCW glycan degradation in the ileal mucosa. Furthermore, we evidenced three GH genes (encoding the GH5 of IL_C5, GH9 of IL_D9/C4 and GH16 of IL_F5/B6/B1/D12) involved in degradation of PCW polysaccharides, which are also part of PULs from the *B. uniformis* type-strain. We thus provided the first experimental validation of GH activities in *B. uniformis*, supporting by the type-strain predicted PULs.

The eighth *Bacteroides* clone (IL_A3) is related to *B. cellulosilyticus*. Up to date, only two different strains, CRE21 and WH2, have been described from this species, isolated from the human gut microbiota^35^,^45^. Even if frequent in human gut metagenomes^4^, *B. cellulosilyticus* has been poorly studied. However, McNulty and collaborators^35^ demonstrated a huge arsenal of genes involved in carbohydrate utilization in *B. cellulosilyticus* WH2 conferring to the strain a competitive advantage for various nutrient selections in the gut. Using functional metagenomic, we evidenced one insert originated from the ileal mucosa which is affiliated to this species, suggesting that *B. cellulosilyticus* could establish in the small bowel.

Our last three clones IL_B1, IL_B5 and IL_H1 were assigned to Clostridiales and related to different *Eubacterium* species. Because of synteny rupture in the three hit clone insert sequences, we cannot afford to affiliate these clones to one or another species (especially for IL_B5) or to cultivated strains (for IL_B3, IL_H1). We thus propose their belonging to yet undescribed novel *Eubacterium* species or strains.

The three clones contain DNA genomic fragment related to *E. rectale.* This species is described until now as a gut species specialized in starch and fructo-oligosaccharide degradation from dietary fibres, and is a beneficial butyrate-producer microorganism for health maintenance in humans^46^. Our IL_B3 clone bears enzymes for inulin (GH32 and GH91) and PCW polysaccharide (GH5) degradation, supporting hydrolytic function of both substrates. More recently, *E. rectale* was proposed as a nutritionally highly specialized species developing specific interactions with the host immune system thanks to a flagellar system^47^. A mucosal location may improve these interactions.

Finally, our study provided clue elements to suggest co-housing of fibrolytic *Bacteroides* and *Eubacterium* species in the mucosa-associated microbiota of the ileum.

Functional screening of metagenomic clones with large DNA fragment inserts is a powerful tool to access to large contigs of bacterial genes giving reliable information on their phylogenetic affiliation and predictive functions. However, a particular attention has to be given to short read sequence assembly (from shotgun technology) into large contigs for accurate further analyses. By using home-made supervised pipeline and manual curation, high quality sequence assembly was obtained for the eleven metagenomic inserts and sequences were carefully examined for their predicted origins. The lack of unique genome affiliation for all the metagenomic inserts and the frequently observed unknown or unreliable taxonomic affiliation in several clones (especially in IL_C5, IL_B5/B3/H1) led us to propose these inserts as part of yet undescribed strains or species that are almost located in the distal part of the small bowel. In addition, half of the whole CDS could not be annotated because of poor (or no) similarity to known proteins, which is coherent with the great number of unclassified (UC) proteins in the eggNOG analysis. The large number of predicted hypothetical proteins found in our metagenomic inserts thus emphasized genomic sequences with poorly understood encoded functions which could however contribute to the metabolic and physiological requirements of the ileal mucosa environment.

Metagenomic inserts are distinct from cultivated strain genomes in a different manner. IL_C5 and IL_B5 inserts are a juxtaposition of sequences originating from almost two different known species (see Table S2), with distinct GC contents (ΔGC of 9% and 5%, respectively, not shown). Four metagenomic inserts (IL_F5, IL_A3, IL_B5, IL_B3) contains remnants of genetic mobile elements (GMEs), mainly integrative and conjugative elements (ICEs). ICEs were located nearby proteins affiliated to species different from those of the remaining sequences, suggesting horizontal gene transfer (HGT) events which contribute to phylogenetic divergence, as already observed in fecal metagenomic inserts encoding GH enzymes^23^,^48^. HGT is widespread among gut bacteria like *Faecalibacterium prausnitzii, Bacteroides* and *Parabacteroides* species^49^, *Enterobacteriaceae*^50^ and *Lachnospiraceae*^51^. It plays a key role in the evolution of bacteria with diversification and speciation, but also provides the ability to bacteria to exploit new environments^50^ and adapt to new ecological niches.

Our study demonstrated the presence in the ileum of bacterial genomes encoding a diversity of CAZymes which may confer to the mucosa-associated microbiota large substrate specificity for PCW polysaccharide degradation. Indeed, the global functional picture of our eleven clones (illustrated in Fig. 5B) uncovered 25 distinct CAZymes (GH, GT and CE) from 17 different protein families. It represents for the mucosa-associated bacteria a strong enzymatic potential to disrupt glycans composed of different sugars monomers and O-glycosidic bonds entering the GI tract as dietary fibres. Among the 25 CAZymes, several GH corresponds to the expected ones regarding the nature of substrates used in our study. In the case of the large GH5 and GH9 families, the former comprises members demonstrating over 20 known specificities^52^. Other GH families like GH2, GH3, GH16, GH94 and GH127 complete the necessary enzymes to disrupt PCW fibres.

In addition, GH families involved in the degradation of storage glycans were also evidenced, like GH32 and GH91 which are dedicated to the degradation of fructose-based oligo- and polysaccharides. The GH13, GH31 and GH97 are specific to α-linked glucose polymers like starch, which is with PCW polysaccharides a major source of carbohydrates delivered in the intestine and an easily fermented substrate for Bacteroidetes^53^. Finally, the presence of GH20 may illustrate the Bacteroidetes affinity for host mucosal glycans^54^.

The multiplicity of GH families identified in this study supports the hypothesis that enzymatic equipments from bacteria of the ileal mucosa are adapted to the catabolism of various carbohydrate dietary fibres. Even though our *Bacteroides* and Clostridiales metagenomic clones are concerned, this poly-specificity is more documented for *Bacteroides* species^54^.

Focusing on PCW dedicated GH, the *Bacteroides* IL_C5 and the Firmicutes IL_B5/B3/H1 bear GH5 enzymes involved in mix-linkage glucan and xylan degradation, and classified in sub-families GH5_2 and GH5_7 which contain mainly extracellular endoglucanases, and β-mannosidases, respectively^52^. The GH9 of IL_D9 and IL_C4 belongs to an endoglucanase family^55^, two of them (IL_D9_CDS 14/IL_C4_CDS 1 and IL_C4_CDS 10) are distant relatives of the previous described GH9. Among the evidenced GHs, the GH16 is of particular interest. Its membership to family GH16 predicts an activity against β-glucan (Table 1), as observed during our screenings. However, transposition assays in our study clearly associated the GH16 encoding genes to the xylanase activity of the *Bacteroides* corresponding clones. Substrate specificities of the GH from the *B uniformis* ATCC 8492 PUL21, including the non classified GH, thus require an in depth characterization.

GHs from families 2, 3, 94 and 127 evidenced in the *Bacteroides* clones provide the mucosa-associated bacteria the capacity to cleave β-linked oligosaccharides or side chains. Furthermore, the presence of PULs in these clones could confer to the corresponding strains efficient enzymatic systems dedicated to the breakdown of mixed-linkage glucans and xylans, as already observed in *B. thetaiotaomicron* and *B. ovatus*^29^. These PULs comprise both endo and exo-acting enzymes and provide an efficient outer-membrane system for bacteria to hydrolyze polysaccharides and release simple sugars for its own metabolism or for interrelations with the host^29^. These enzymatic features, associated with GH activities exhibited in the positive clones, oligosaccharide released and transposon mutagenesis (Fig. 4) strongly support the hypothesis that bacteria associated to the ileal mucosa bear diversified functional equipments to hydrolyze PCW polysaccharides and internalize efficiently sugar end-products.

Human ileal and fecal microbiota were rarely compared at the functional level^1^,^18^. In addition, ileal metagenomes from large cohorts are desperately missing to estimate gene prevalence and abundance in this intestinal niche. Our study provides the first evidence that genomic sequences encoding PCW polysaccharide GH and gene clusters involved in complex glycan metabolism commonly found in fibrolytic species are present in both ecosystems, but also pointed out several clusters of genes which are only associated to the ileal mucosa-associated microbiota. Indeed, by searching for homologous genes in fecal metagenomes from the 9.9 M gene MetaHit integrated catalog and estimating their prevalence in 1,267 individuals, four clusters of genes from our ileal mucosa inserts, involved in carbohydrate metabolism, were identified with a low prevalence in fecal microbiota. They encode *Bacteroides* GH from families 16, 127 and the unknown GH family, as well as the SusCD-like proteins usually involved in PUL. They also contain *E. rectale* GH from families 32 and 91, combined with ABC-transporters. Combination of GH and oligosaccharide transporters has recently been described in *E. rectale* and *Roseburia* species as gram positive PUL (gpPUL)^47^. Such gene clusters are dedicated to carbohydrate utilization like in *Bacteroides* but their organization is nevertheless quite different. Both PUL and gpPUL confer to the concerned bacterial species nutritional specialization and an efficient way to rapidly internalize fermentable oligosaccharides. Such systems confer ecological advantage to bacteria which quickly adapt and respond to varying carbohydrate availability compared to other microorganisms.

Finally, genes from the low prevalence clusters are not part of the fecal dominant microbiota but should be associated to major bacterial strains of another intestinal ecosystem located in the proximal bowel and especially in the ileal mucosa.

In conclusion, even though assessment of fibrolytic bacteria associated to the mucosa of the human ileum has to be deepened, it should be emphasized that microbiota from the ileum mucosa encompasses the potential for fiber degradation. This is an important finding regarding the organization of the microbial trophic network in the proximal bowel, but also because of the intimate interactions with the host for energy harvest and health benefits.

This potential could be partly supported by strains undescribed in fecal bacteria we proposed as dominant mucosa-associated strains in the ileum. Microbial gene repertoires from ileal microbiota, including mucosa-associated and luminal bacteria, would be necessary such as those for other gastro intestinal sites for more powerful data mining, assorted to the isolation of fibrolytic strains for in-depth functional studies.

## Methods

### Bacterial strains and culture conditions

*Escherichia coli* EC100 cells (F^−^ mcrA Δ(mrr-hsdRMS-mcrBC) Φ80dlacZΔM15 ΔlacX74 recA1 endA1 araD139 Δ(ara, leu)7697 galU galK λ^−^ rpsL (Str^R^) nupG genotype) and *E. coli* EC100-T1^R^ cells (F^–^ mcrA ∆(mrr-hsdRMS-mcrBC)ϕ80dlacZ∆M15 ∆lacX74 recA1 endA1 araD139∆(ara, leu)7697galU galK λ–rpsL nupG tonA genotype) from Epicentre Technologies (Madison, WI, USA) are used in this study. pEpiFOS-5 is a chloramphenicol resistant fosmid cloning vector (Cm^R^).

Metagenomic clones, as well as EC100 *E. coli* cells containing only the pEpiFOS-5 vector, were cultivated at 37 °C in Luria Bertani (LB) broth (BD Difco^TM^, New Jersey) containing 12.5 μg/mL chloramphenicol (Cm) (LB-Cm medium), or 12.5 μg/mL chloramphenicol plus 50 μg/mL kanamycin (Kan) (LB-Cm-Kan medium) when used for transposon mutagenesis. Two successive cultures were performed for 24 h and 18 h, respectively, in LB broth (5 ml) with appropriate antibiotics, at 37 °C with 160 rpm stirring, before performing any experiment.

### Functional screening of the metagenomic library

The library produced from the ileal mucosa-associated microbiota is an EC100 *E. coli* pEpiFOS-5 fosmidic library (Epicentre Technologies) constructed with microbial DNA extracted from the healthy distal part of the ileum of a colorectal cancer patient biopsy, as previously described^33^. Twenty thousand clones with large DNA inserts ranging from 30 to 40 Kb and covering 800 Mb of metagenomic sequences constitute the library.

Functional screening for carboxymethylcellulase, xylanase, β-glucanase and lichenase activities was performed as follow: after growth in LB-Cm broth in 96-wells microplates, the 20,000 metagenomic clones were spotted with propylene replicators on the surface of 13cm × 9 cm OmniTray single-well plates. These plates contained two layers of medium: a first one of 25 mL LB agar medium with 12.5 μg/μL Cm and a second one, spread out in overlay, of 20 mL LB agar medium with 12.5 μg/μL Cm, 0.5 mM MgCl_2_, 0.5 mM CaCl_2_. and 0.5% (wt/v) polysaccharide. Added polysaccharide was medium viscosity carboxymethylcellulose (CMC), oat spelt (OS) xylan (both from Sigma-Aldrich, France), β-glucan or lichenan (both from Megazyme, Wicklow, Irlande). Both layers were buffered to pH 6.8 with 50 mM sodium phosphate (NaPi) buffer.

Cells grew into colonies at 37 °C during 3 days, then colonies were removed from the agar surface. The plates were then flooded with 10 mL of 0.1% Congo red for 30 minutes and washed off three times with 10 mL of 1 M NaCl for 15 minutes. The red dye strongly interacts with polysaccharides and clear zones appear around positive colonies. If the observed halos were comparable to those of the negative control EC100 *E. coli/*pEpiFOS-5, results are considered negative.

### Protein samples and agar well plate enzymatic assays

To prepare protein samples, each metagenomic clone was cultivated overnight at 37 °C in 40 mL LB-Cm and then centrifuged at 5,000 × g for 15 min at 4 °C, in order to obtain 50-fold concentrated fractions. Supernatants were transferred under sterile conditions in a Spin-X UF 10 K concentrator (manufacturer’s protocol, Corning B.V., Amsterdam) and pellets were resuspended in 800 μL of sterile 50 mM NaPi buffer. Mechanical cell lysis was performed on pellets, after adding glass beads (Sigma Aldrich, Saint-Quentin-Fallavier, France), at 40 s at 5 m.sec^−1^ with a FastPrep^®^−24 (MP-Biomedicals, NY, USA).

Agar-well plate enzymatic assays were performed to detect endoglucanase, xylanase, β-glucanase, lichenase and xyloglucanase activities of 50-fold concentrated extracellular or cell-associated proteins. Samples were loaded (80 μL/well) in Omnitray plates containing 45 mL of LB-NaPi supplemented with 0.5% CMC, OS xylan, β-glucan, lichenan or xyloglucan (from Megazyme, Wicklow, Irlande), 12.5 μg/μL Cm, 0.5 mM MgCl_2_ and 0.5 mM CaCl_2_. The plates were incubated for 24 hours at 37 °C and stained with Congo red as described for the functional screening. Clear halos revealed substrate degradation around wells containing polysaccharidic enzymes. Here again, intensity of the halo comparable to those of the EC100 *E. coli/*pEpiFOS-5 control is considered as negative.

### Analysis of end products by Thin Layer Chromatography (TLC)

Twenty microliters of a 0.5% polysaccharide-NaPi solution (wt/v) were incubated with 20 μL of 50X concentrated cell-associated proteins of each active clone for 3 hours at 37 °C. Ten microliters of the mix were plotted on a baseline at 1 cm from the bottom of a thin-layer silica gel sheets (Merck, TLC Silica Gel 60 F254). Sugars were separated after a double 30-minute migration in one dimension as previously described^56^, using butanol-acetic acid-water (8:10:1.5) as separative solution and 0.5% thymol, 5% sulfuric acid in ethanol for revelation solution. Sugars released from CMC, OS xylan, β-glucan and lichenan were qualitatively determined by comparison with different oligosaccharides used as standards (0.5% each in NaPi solution (wt/v)) and loaded on the TLC (1 μL each). The pEpiFOS-5/EC100 *E. coli* protein samples were used as negative control, and the non-digested polysaccharides were added on TLC to ensure their integrity during the experiment.

### DNA isolation and transposon mutagenesis

The Macherey-Nagel Nucleobond PC20 kit (MN, Hoerdt, France) was used to isolate fosmidic DNA using the manufacturer’s protocol. DNA was quantified using a NanoDrop N-1000 spectrophotometer (NanoDrop Technologies) and quality assessment was performed with 0.6% (w/v) ethidium bromide-stained agarose electrophoresis.

Transposon mutagenesis was carried out on fosmidic DNA using the EZ-Tn5 <KAN2> Insertion Kit and Transformax electro-competent *E. coli* EC100-T1^r^ cells (Epicentre Technologies, Madison, USA) following the manufacturer’s protocol. Inactivated clones for polysaccharidic activity were then identified by plating the kanamycin-resistant transposon insertion clones on LB-Cm-Kan agar plates supplemented with the appropriate polysaccharide. Sanger sequencing (Beckman Coulter Genomics, United Kingdom) was performed to localize the transposon insertion site in metagenomic insert DNA of inactivated clones, using the FP-1 and/or RP-1 primers supplied in the kit.

### Sequencing and annotation of metagenomic DNA inserts

The sequencing of fosmid inserts was performed using 454 GS FLX titanium (454 Life Sciences, Brandford, CT) or MiSeq (Illumina, San Diego, CA) systems by the INRA Genomic and Transcriptomic platform (GET Genotoul, Auzeville, France). Read assemblies were performed using SPAdes version 3.5.0^57^ to obtain contig sets. Down-sampling to 20,000 reads, corresponding to approximately 100X genome coverage, was applied for MiSeq inserts sequencing. From these sets, contigs corresponding to the awaited length and sequencing depth were extracted, generally one per fosmid. Each contig was then vector cleaned using crossmatch (http://www.phrap.org/phredphrapconsed.html) and reorganized in a unique sequence. The sequences have been validated through read re-mapping, no null coverage zone was observed. The full set of metagenomic insert sequences has been deposited in the European Nucleotide Archive (ENA) under the accession numbers LT674122-LT674132 (http://www.ebi.ac.uk/ena/data/view/LT674122-LT674132).

The taxonomic assignment of full metagenomic insert sequences was based on nucleic sequence similarity using the microbial nucleotide Basic Local Alignment and Search Tool (Blast) at NCBI facilities^58^ with either representative genomes or all complete genomes, as well as by performing classic BlastN using the non-redundant nucleotide database nt (identity percentage > 97%).

Coding DNA sequences (CDS) were predicted using FramePlot^59^ and PROKKA^60^ packages. Most of CDS were identical except that PROKKA did not detect the two incomplete CDS at the 5’ extremity of the IL_C4 and IL_B3 clones. The sequences were manually corrected for several frameshifts in IL_B6, IL_C4, IL_F5 and IL_D12. Annotations of putative proteins were performed by homology searches using the Blast facilities against Reference Protein Sequences database and were validated using tBlastN with the MetaHit 9.9 M gene catalogue. Best hits are summarized in Supplementary Table S2.

EggNOG assignment was performed with 251 predicted proteins from hit clone CDS, including non-redundant proteins for either IL-F5/B6/B1/D12 or IL-D9/C4, using BlastP with the eggNOG 4.0 database^61^ (E-value ≤ 10^−7^, identity percentage ≥ 80%, best hit chosen). We assigned eggNOGs to members of the non-supervised orthologous groups of Bacteria, Bacteroidetes, Firmicutes, Bacteroidia and Bacilli. The functional category was assigned for 145 proteins with a BlastP result. For the remaining unclassified (UC) CDS encoded proteins, 56% were short predicted proteins (<150aa). For proteins with multiple eggNOG assignation, we have chosen the consensus category when possible, otherwise, the first one. The radial plot was made with R radial.plot function of the plotrix library^62^.

### CAZYme annotation and PUL prediction

Carbohydrate related enzymes encoding genes were identified by Blast analysis of the predicted ORFs against the functional modules of all CAZyme groups (GH, PL, CE, GT and CBM) included in the Carbohydrate Active Enzyme database (http://www.cazy.org) using a cut-off E-value of 7.10^−6^ followed by visual inspection and alignment with known CAZy families^27^ to validate the prediction with human curation. The presence of Polysaccharide-Utilization Locus (PUL) in metagenomic insert sequences was determined using Blast against protein datasets for species in the Polysaccharide-Utilization Locus Database (PULDB) at http://www.cazy.org/PULDB/63.

### Gene abundance in metagenomes and reference catalogue

Genes homologs to the 251 non-redundant CDS of the eleven positive clones were searched in ileal metagenomic sequences (raw reads or assembled genes) using the very limited available data from microbiota of ileal effluents from ileostomized individuals^1^,^36^, and metagenomic clones from the mucosa of ileum^23^. Short reads were aligned on all CDS with Bowtie 0.12.7^64^ (read length: 100nt, 3 mismatches allowed). Sequences of Cecchini^23^ and Zoetendal^1^ metagenomic clones were blasted against the 251 CDS of our eleven clones (BlastN, E-value < 1e-05, identity percentage > 80% and alignment length > 30%).

The 52 non-redundant CDS encoded proteins from the eleven positive clones comprising CAZymes, SusCD-like and eggNOG [G] proteins were also searched in the translated MetaHIT integrated catalogue of 9.9 million human gut microbiota reference genes, constructed using 1,267 fecal samples from European, Chinese and U.S. individuals^6^. Homologs were selected using BlastP (*E-*value = 1e-05, identity percentage ≥ 80% and alignment length ≥ 90%). Taxonomic annotation of the hits found in the catalogue was performed on the corresponding genes using BlastN and the NCBI nt and WGS databases.

Heat map was generated using the *momr* R package and the deposited 9.9 M gene frequency matrix (http://meta.genomics.cn/metagene/meta/dataTools). Microbial gene richness was measured by counting the number of genes identified in a given sample using 11 million reads as performed in the original studies^65^.

## Additional Information

**How to cite this article**: Patrascu, O. *et al*. A fibrolytic potential in the human ileum mucosal microbiota revealed by functional metagenomic. *Sci. Rep.* **7**, 40248; doi: 10.1038/srep40248 (2017).

**Publisher's note:** Springer Nature remains neutral with regard to jurisdictional claims in published maps and institutional affiliations.

## Supplementary Material

Supplementary Information

Supplementary Table S2

Supplementary Table S4

Supplementary Table S5

## Figures and Tables

**Table 1:** CAZyme annotation and predictive function of CDS encoding genes from the eleven positive metagenomic clones.

Clone	Taxonomic assignation^d^	Clone activity	CAZyme family	Size (aa)	Putative function	Best hit characterized^e^ (aa percent identity)
IL_C5	*Bacteroides*	CMCase, xylanase, β-glucanase, lichenase	GH5_2GH5_7CE7GH2GH94GH97GH2GH127GH127	326430427814828671831696788	endoglucanaseβ-1,4 mannosidase, β-mannanasedeacetylaseβ-galactosidasecellobiose phosphorylaseα-galactosidaseβ-galactosidaseβ-L-arabinofuranosidaseβ-L-arabinofuranosidase	AGL50932.1 endoglucanase (PMID = 25022521) (63%)AAS19695.1 β-mannosidase (http://www.prozomix.com/products/view?product=132) (44%)AAD35171.1 acetyl esterase (PMID = 22411095) (30%)BAJ61032.1 β-galactosidase (PMID = 25473598) (34%)AAL67138.1 cellobiose phosphorylase (PMID = 22102229) (65%)AAO76978.1 α-galactosidase (PMID: 18848471) (46%)AAP86764.1 β-galactosidase (PMID: 15153767) (44%)BAK79118.1 β-L-arabinofuranosidase (PMID = 24385433) (30%)BAK79118.1 β-L-arabinofuranosidase (PMID = 24385433) (32%)
IL_D9^a^	*Bacteroides*	CMCase, xylanase, β-glucanase, lichenase	GH9GH31GH9	842791576	endoglucanase/xyloglucanaseα-glucosidase/α-xylosidaseendoglucanase/xyloglucanase	CAA28255.1 endoglucanase (PMID = 9335164) (22%)AAO75446.1 α-glucosidase (PMID = 23036359) (41%)WP_004298437.1 endo-xyloglucanase (PMID = 24463512) (70%)
IL_C4^a^	*Bacteroides*	CMCase, xylanase, β-glucanase, lichenase	GH9GH31GH9GH9CE4fragment	842791576844218	endoglucanase/xyloglucanaseα-glucosidase/α-xylosidaseendoglucanase/xyloglucanaseendoglucanase/xyloglucanasedeacetylase	CAA28255.1 endoglucanase (PMID = 9335164) (22%)AAO75446.1 α-glucosidase (PMID = 23036359) (41%)WP_004298437.1 endo-xyloglucanase (PMID = 24463512) (70%)ACL75133.1 endoglucanase (PMID = 24451379) (22%)AAP10549.1 peptidoglycan N-acetylglucosamine deacetylase (PMID:15961396) (34%)
IL_F5^b^	*Bacteroides*	Xylanase, β-glucanase, lichenase	GH13GH97GH3GHncGH16	616717750430314	α-glucosidaseα-glucosidaseβ-glucosidaseunknownβ-1,3-glucanase	AAO78809.1 neopullulanase (PMID = 8955399) (52%)AAC44671.1 α-glucosidase (PMID = 18981178) (70%)AEW47953.1 β-glucosidase (PMID = 23906845) (72%)no hitAAC69707.1 laminarinase (PMID = 7925416) (41%)
IL_B6^b^ and IL_B1^b^	*Bacteroides*	Xylanase, β-glucanase, lichenase	GH3GHncGH16GT2	750430314317	β-glucosidaseunknownβ-1,3-glucanaseβ-glycoside transferase	AEW47953.1 β-glucosidase (PMID = 23906845) (72%)no hitAAC69707.1 laminarinase (PMID = 7925416) (41%)AAC75314.1 undecaprenyl-phosphate-L-Ara4FN transferase (PMID = 17928292) (32%)
IL_D12^b^	*Bacteroides*	Xylanase, β-glucanase, lichenase	GH97GH3GHncGH16GT2	717750430314317	α-glucosidaseβ-glucosidaseunknownβ-1,3-glucanaseβ-glycoside transferase	AAC44671.1 α-glucosidase (PMID = 18981178) (70%)AEW47953.1 β-glucosidase (PMID = 23906845) (72%)no hitAAC69707.1 laminarinase (PMID = 7925416) (41%)AAC75314.1 undecaprenyl-phosphate-L-Ara4FN transferase (PMID = 17928292) (32%)
IL_A3	*Bacteroides*	Xylanase, β-glucanase, lichenase	GH97GH3GH16GH20	717750314814	α-glucosidaseβ-glucosidaseβ-1,3-glucanaseβ-N-acetylglucosaminidase	AAC44671.1 α-glucosidase (PMID = 18981178) (70%)AEW47953.1 β-glucosidase (PMID = 23906845) (72%)AAC69707.1 laminarinase (PMID = 7925416) (41%)BAD48481.1 β-N-acetylglucosaminidase (PMID = 22449996) (40%)
IL_B5^c^	Clostridiales	CMCase, β-glucanase, lichenase	GH5_2	386	endoglucanase	ABA42185.1 endoglucanase (PMID = 17216439) (47%)
IL_B3^c^	Clostridiales	CMCase, β-glucanase, lichenase	GH32GH91GH5_2	308453386	β-fructosidaseDFA IIIaseendoglucanase	AAC33123.1 invertase (PMID = 10446718) (40%)BAD06469.1 di-fructofuranose 1,2′:2,3′ dianhydride hydrolase (PMID = 16233453) (67%)ABA42185.1 endoglucanase (PMID = 17216439) (47%)
IL_H1^c^	Clostridiales	CMCase, β-glucanase, lichenase	GH5_2	386	endoglucanase	ABA42185.1 endoglucanase (PMID = 17216439) (47%)

nc: non classified.

^a^clones partially redundant. The GH9, GH31 and GH9 of IL_D9 are identical to the first three GH of IL_C4.

^b^clones partially redundant. Proteins from the same CAZyme family are identical in the corresponding clones.

^c^clones partially redundant. The GH5_2 enzymes are identical.

^d^Order, Family or genus assigment, according to BlastP analyses using the database Reference Protein Sequences (ref_seq_protein).

^e^Genbank accession number and PubMed-indexed for MEDLINE (PMID) are given for each best hit.
