# Supplementary material for: A fibrolytic potential in the human ileum mucosal microbiota revealed by functional metagenomic
Source: Sci Rep. 2017 Jan 16;7:40248. doi: 10.1038/srep40248 (PMC5238381; doi:10.1038/srep40248)
Supplement: Supplementary Information [file srep40248-s1.pdf]

SUPPLEMENTARY INFORMATION

A fibrolytic potential in the human ileum mucosal microbiota revealed by functional metagenomic.

Orlane Patrascu[1], Fabienne Béguet-Crespel[1], Ludovica Marinelli[1], Emmanuelle Le Chatelier[2], Anne-Laure Abraham[1], Marion Leclerc[1], Christophe Klopp[3], Nicolas Terrapon[4,5], Bernard Henrissat[4,5,6], Hervé M Blottière[1,2], Joël Doré[1,2] & Christel Béra-Maillet[1*]

[1] Micalis Institute, INRA, AgroParisTech, Université Paris-Saclay, 78350 Jouy-en-Josas, France

[2]Metagenopolis, INRA, 78352 Jouy-en-Josas, France

[3]Plate-forme bio-informatique Genotoul, Mathématiques et Informatique Appliquées de Toulouse, INRA, Castanet-Tolosan, France

[4]CNRS UMR 7257, Aix-Marseille University, 13288 Marseille, France

[5]INRA, USC 1408 AFMB, 13288 Marseille, France

[6]Department of Biological Sciences, King Abdulaziz University, Jeddah, Saudi Arabia

* Corresponding author: Dr Christel Béra-Maillet

Mailing address: UMR1319 MICALIS, INRA, Domaine de Vilvert, F-78352 Jouy-en-Josas cedex 9, France; phone: +33 1 3465 2784; e-mail: christel.maillet@inra.fr

SUPPLEMENTARY FIGURE AND TABLE LEGENDS

Supplementary Figure S1: Radial plot of EggNOG functional category assignments of the non-redundant CDS for each clone considered individually (see Fig. 3 for the legend).

Supplementary Table S1: BlastP output for EggNOG assignments of the non redundant CDS of the eleven clones.

Supplementary Table S2: All CDS connections, taxonomic affiliations and annotations between the eleven metagenomic clones.

Supplementary Table S3: Putative activities of the evidenced Glycoside Hydrolase (GH) and Carbohydrate Esterase (CE) families in the eleven positive clones.

Supplementary Table S4: *in silico* analysis of GH homologous genes in available metagenomes from the ileum intestinal site.

Supplementary Table S5: BlastP output of homologous genes from the MetaHit 9.9M reference catalogue to the positive clone CDS-encoding genes.

SUPPLEMENTARY FIGURE

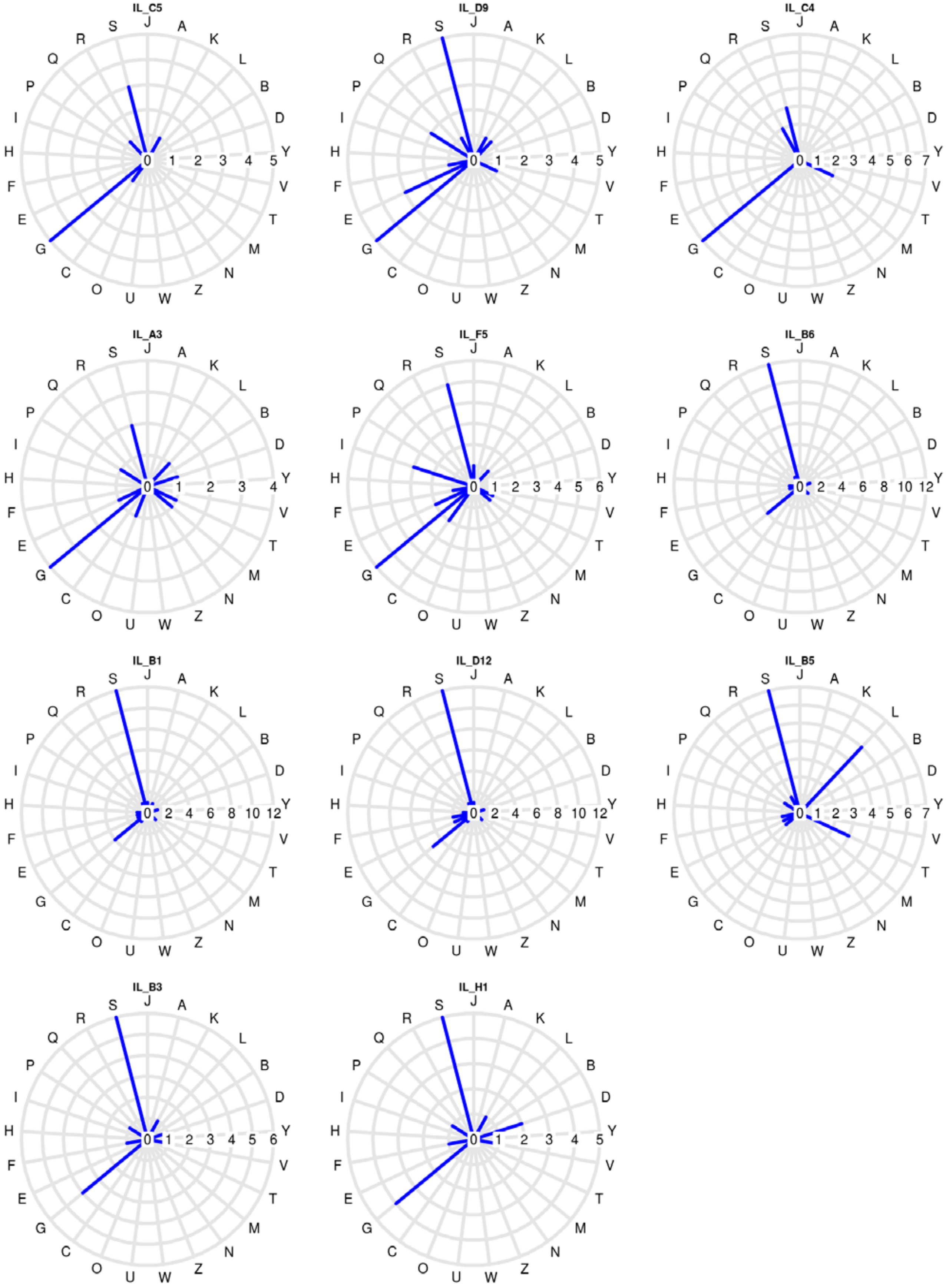

Figure S1: Radial plot of EggNOG functional category assignments of the non-redundant CDS for each clone considered individually (see Fig. 3 for the legend).

SUPPLEMENTARY TABLES

Table S1: BlastP output for EggNOG assignments of the non redundant CDS of the eleven clones.

CDS	Hit name[a]	identity (%)	alignment le	mismatches	gap opening	q. start	q. end	s. start	s. end	e-value	bit score	EggNOGG category[b]	1st assignati	1st letter	2nd assignati	2nd letter	3rd assignati	3rd letter
IL_C5_CDS_1	585543.HMPREF0969_02168	99.06	319	3	0	8	326	1	319	0.0	662	G	bactNOG228	G	bctoNOG031	G	NA	NA
IL_C5_CDS_2	693979.Bache_0234	86.44	413	55	1	17	429	17	428	0.0	755	G	bactNOG246	G	NA	NA	NA	NA
IL_C5_CDS_3	585543.HMPREF0969_02166	98.83	427	5	0	1	427	1	427	0.0	851	Q	bacteNOG04	Q	bactNOG109	Q	bctoNOG040	Q
IL_C5_CDS_4	585543.HMPREF0969_02165	100.00	295	0	0	3	297	1	295	0.0	616	K	bacteNOG01	K	bactNOG313	K	bctoNOG017	K
IL_C5_CDS_5	585543.HMPREF0969_02164	91.40	814	70	0	1	814	1	814	0.0	1546	G	bacteNOG04	G	bactNOG015	G	bctoNOG043	G
IL_C5_CDS_6	585543.HMPREF0969_02162	98.91	828	9	0	1	828	1	828	0.0	1648	G	bacteNOG00	G	bactNOG054	G	bctoNOG041	G
IL_C5_CDS_7	585543.HMPREF0969_02161	99.40	334	2	0	1	334	1	334	0.0	669	C	bacteNOG01	C	bactNOG228	C	bctoNOG029	C
IL_C5_CDS_8	585543.HMPREF0969_02160	99.55	671	3	0	1	671	1	671	0.0	1400	G	bactNOG011	G	bctoNOG006	G	NA	NA
IL_C5_CDS_9	585543.HMPREF0969_02159	100.00	212	0	0	1	212	1	212	8,00E-144	405	S	bacteNOG02	S	bactNOG868	S	bctoNOG110	S
IL_C5_CDS_10	585543.HMPREF0969_02158	100.00	209	0	0	1	209	1	209	8,00E-140	395	S	bacteNOG02	S	bactNOG808	S	bctoNOG110	S
IL_C5_CDS_14	449673.BACSTE_03758	98.43	830	13	0	1	830	1	830	0.0	1709	S	bacteNOG04	S	bactNOG029	S	bctoNOG029	S
IL_D9_CDS_1	585543.HMPREF0969_01720	100.00	139	0	0	1	139	576	714	3,00E-94	293	K	bacteNOG00	K	bactNOG002	K	bctoNOG001	K
IL_D9_CDS_2	585543.HMPREF0969_01719	100.00	135	0	0	1	135	24	158	2,00E-97	283	R	bacteNOG02	R	bactNOG117	R	bctoNOG143	R
IL_D9_CDS_3	585543.HMPREF0969_01718	98.41	315	5	0	1	315	1	315	0.0	552	E	bacteNOG01	E	bactNOG004	E	bctoNOG000	E
IL_D9_CDS_4	585543.HMPREF0969_01717	100.00	299	0	0	1	299	1	299	0.0	596	E	bacteNOG00	E	bactNOG030	E	bctoNOG068	E
IL_D9_CDS_5	585543.HMPREF0969_01716	100.00	400	0	0	1	400	1	400	0.0	831	L	bacteNOG00	L	bactNOG003	L	bctoNOG002	L
IL_D9_CDS_6	585543.HMPREF0969_01715	98.38	741	12	0	5	745	1	741	0.0	1494	E	bacteNOG00	E	bactNOG004	E	bctoNOG005	E
IL_D9_CDS_7	585543.HMPREF0969_01714	99.29	424	3	0	1	424	1	424	0.0	856	F	bacteNOG00	F	bactNOG001	F	bctoNOG007	F
IL_D9_CDS_8	585543.HMPREF0969_01713	100.00	331	0	0	1	331	1	331	0.0	634	S	bacteNOG03	S	bactNOG101	S	bctoNOG127	S
IL_D9_CDS_9	585543.HMPREF0969_01712	100.00	159	0	0	1	159	1	159	3,00E-112	321	S	bacteNOG02	S	bctoNOG118	S	NA	NA
IL_D9_CDS_10	585543.HMPREF0969_01711	98.66	224	3	0	1	224	2	225	3,00E-165	461	S	bacteNOG02	S	bactNOG237	S	bctoNOG032	S
IL_D9_CDS_11	585543.HMPREF0969_01710	99.33	300	2	0	1	300	1	300	0.0	580	P	bacteNOG02	P	bactNOG239	P	bctoNOG070	P
IL_D9_CDS_12	585543.HMPREF0969_01709	99.63	269	1	0	1	269	1	269	0.0	550	P	bacteNOG01	P	bactNOG098	P	bctoNOG039	P
IL_D9_CDS_13	585543.HMPREF0969_01708	99.51	607	3	0	23	629	1	607	0.0	1228	S	bactNOG359	S	NA	NA	NA	NA
IL_D9_CDS_14	585543.HMPREF0969_01707	94.77	842	44	0	1	842	1	842	0.0	1681	G	bactNOG172	G	bctoNOG048	G	NA	NA
IL_D9_CDS_15	585543.HMPREF0969_01706	81.91	398	72	0	14	411	1	398	0.0	700	G	bactNOG036	G	NA	NA	NA	NA
IL_D9_CDS_18	585543.HMPREF0969_01703	97.09	791	23	0	1	791	1	791	0.0	1591	G	bacteNOG01	G	bactNOG013	G	bctoNOG011	G
IL_D9_CDS_19	585543.HMPREF0969_01702	87.60	1306	159	1	1	1306	1	1303	0.0	2355	T	bactNOG000	T	NA	NA	NA	NA
IL_D9_CDS_20	585543.HMPREF0969_01701	96.24	559	21	0	1	559	1	559	0.0	1060	G	bactNOG052	G	bctoNOG054	G	NA	NA
IL_D9_CDS_21	585543.HMPREF0969_01700	96.05	633	25	0	1	633	1	633	0.0	1274	S	bactNOG108	S	NA	NA	NA	NA
IL_C4_CDS_9	585543.HMPREF0969_01699	94.90	412	21	0	30	441	6	417	0.0	824	G	bactNOG036	G	NA	NA	NA	NA
IL_C4_CDS_10	585543.HMPREF0969_01698	98.22	844	15	0	1	844	1	844	0.0	1662	G	bactNOG172	G	bctoNOG048	G	NA	NA
IL_C4_CDS_12	457424.BFAG_03926	88.00	375	45	0	1	375	1	375	0.0	705	R	bactNOG397	R	bctoNOG033	R	NA	NA
IL_C4_CDS_15	585543.HMPREF0969_01691	99.73	1114	3	0	1	1114	1	1114	0.0	2192	S	bacteNOG00	S	bactNOG054	S	bctoNOG024	S
IL_C4_CDS_16	585543.HMPREF0969_01690	99.54	218	1	0	1	218	1	218	2,00E-163	456	G	bactNOG946	G	bctoNOG097	G	NA	NA
IL_C4_CDS_19	585543.HMPREF0969_01687	99.05	210	2	0	1	210	1	210	4,00E-153	429	T	bactNOG159	T	bctoNOG122	T	NA	NA
IL_C4_CDS_20	585543.HMPREF0969_01686	99.76	410	1	0	1	410	1	410	0.0	755	S	bacteNOG01	S	bactNOG353	S	bctoNOG075	S
IL_C4_CDS_21	585543.HMPREF0969_01685	100.00	267	0	0	1	267	1	267	0.0	551	R	bacteNOG01	IQ	bactNOG012	R	bctoNOG025	IQ
IL_A3_CDS_7	483216.BACEGG_03755	92.33	404	31	0	1	404	1	404	0.0	790	L	bacteNOG03	L	bactNOG028	L	bctoNOG004	L
IL_A3_CDS_8	471870.BACINT_04166	97.35	718	19	0	1	718	1	718	0.0	1448	G	bacteNOG00	S	bactNOG011	G	bctoNOG006	G
IL_A3_CDS_9	763034.HMPREF9446_00608	81.90	105	19	0	2	106	42	146	4,00E-40	138	S	bacteNOG03	S	bactNOG247	D	bctoNOG154	S
IL_A3_CDS_10	471870.BACINT_04168	98.83	426	5	0	1	426	1	426	0.0	815	G	bacteNOG00	G	bactNOG005	G	bctoNOG007	G
IL_A3_CDS_11	471870.BACINT_04169	93.60	750	48	0	1	750	1	750	0.0	1418	G	bactNOG024	G	NA	NA	NA	NA
IL_A3_CDS_16	585543.HMPREF0969_00341	86.76	1065	125	4	20	1084	19	1067	0.0	1834	P	bactNOG001	P	bctoNOG000	P	NA	NA
IL_A3_CDS_17	471870.BACINT_04174	96.86	700	21	1	74	773	1	699	0.0	1407	S	bacteNOG01	S	bactNOG390	S	bctoNOG044	S
IL_A3_CDS_18	471870.BACINT_04175	99.45	729	4	0	1	729	1	729	0.0	1483	E	bacteNOG04	R	bactNOG228	E	bctoNOG019	E
IL_A3_CDS_19	471870.BACINT_04176	98.15	216	4	0	1	216	1	216	9,00E-159	445	T	bacteNOG00	T	bactNOG237	T	bctoNOG026	T
IL_A3_CDS_20	471870.BACINT_04177	93.49	814	53	0	1	814	1	814	0.0	1559	G	bactNOG054	G	NA	NA	NA	NA
IL_A3_CDS_21	471870.BACINT_04178	99.05	315	3	0	1	315	2	316	0.0	625	O	bacteNOG00	O	bactNOG002	O	bctoNOG003	O
IL_A3_CDS_22	471870.BACINT_04179	92.06	214	17	0	1	214	1	214	7,00E-147	413	M	bactNOG375	M	bctoNOG079	M	NA	NA
IL_A3_CDS_23	471870.BACINT_04180	95.80	834	30	3	1	830	1	833	0.0	1503	D	bacteNOG01	D	bactNOG228	D	bctoNOG012	D

Table S1 (continued):

CDS	Hit name[a]	identity (%)	alignment le	mismatches	gap opening	q. start	q. end	s. start	s. end	e-value	bit score	EggNOGG category[b]	1st assignati	1st letter	2nd assignat	2nd letter	3rd assignati	3rd letter
IL_F5_CDS_2	585543.HMPREF0969_00370	99.78	453	1	0	1	453	1	453	0.0	894	T	bacteNOG00	T	bactNOG001	T	bctoNOG000	T
IL_F5_CDS_3	585543.HMPREF0969_00369	100.00	495	0	0	1	495	1	495	0.0	993	M	bacteNOG01	M	bactNOG051	M	bctoNOG027	M
IL_F5_CDS_4	585543.HMPREF0969_00368	100.00	138	0	0	33	170	1	138	2,00E-95	278	I	bacteNOG02	E	bactNOG958	I	bctoNOG093	I
IL_F5_CDS_5	585543.HMPREF0969_00367	100.00	517	0	0	1	517	1	517	0.0	1070	I	bacteNOG00	I	bctoNOG006	I	NA	NA
IL_F5_CDS_6	585543.HMPREF0969_00366	100.00	310	0	0	1	310	18	327	0.0	606	S	bacteNOG01	S	bactNOG424	S	bctoNOG022	S
IL_F5_CDS_7	585543.HMPREF0969_00365	100.00	144	0	0	1	144	1	144	7,00E-64	198	I	bacteNOG02	I	bactNOG152	S	bctoNOG151	I
IL_F5_CDS_8	585543.HMPREF0969_00364	100.00	386	0	0	1	386	1	386	0.0	665	C	bacteNOG00	C	bactNOG031	C	bctoNOG040	C
IL_F5_CDS_10	585543.HMPREF0969_00362	99.51	616	3	0	1	616	1	616	0.0	1217	G	bacteNOG00	G	bactNOG006	G	bctoNOG011	G
IL_F5_CDS_11	585543.HMPREF0969_00360	100.00	336	0	0	1	336	1	336	0.0	696	G	bacteNOG00	G	bactNOG025	G	bctoNOG041	G
IL_F5_CDS_12	585543.HMPREF0969_00359	98.88	267	3	0	10	276	69	335	0.0	548	S	bacteNOG01	S	bactNOG026	S	bctoNOG022	S
IL_F5_CDS_13	316407.85676327	99.67	304	1	0	121	424	4	307	0.0	637	L	bactNOG365	L	NA	NA	NA	NA
IL_F5_CDS_14	585543.HMPREF0969_00358	100.00	83	0	0	1	83	28	110	6,00E-56	174	J	bacteNOG02	J	bactNOG246	J	bctoNOG135	J
IL_F5_CDS_16	585543.HMPREF0969_00356	99.45	362	2	0	1	362	1	362	0.0	751	E	bacteNOG00	E	bactNOG027	E	bctoNOG020	E
IL_F5_CDS_17	585543.HMPREF0969_00355	99.75	407	1	0	1	407	1	407	0.0	801	E	bacteNOG00	E	bactNOG027	E	bctoNOG016	E
IL_F5_CDS_18	585543.HMPREF0969_00354	100.00	470	0	0	1	470	1	470	0.0	977	F	bacteNOG00	F	bactNOG007	F	bctoNOG005	F
IL_F5_CDS_20	585543.HMPREF0969_00352	99.64	278	1	0	36	313	16	293	0.0	588	C	bactNOG996	C	NA	NA	NA	NA
IL_F5_CDS_21	585543.HMPREF0969_00351	99.58	717	3	0	1	717	4	720	0.0	1496	G	bacteNOG00	S	bactNOG011	G	bctoNOG006	G
IL_F5_CDS_23	585543.HMPREF0969_00349	100.00	125	0	0	1	125	1	125	2,00E-56	177	S	bacteNOG03	S	bactNOG253	S	bctoNOG154	S
IL_F5_CDS_24	585543.HMPREF0969_00348	98.20	222	4	0	1	222	1	222	4,00E-161	450	S	bactNOG119	S	NA	NA	NA	NA
IL_F5_CDS_25	585543.HMPREF0969_00347	99.77	429	1	0	1	429	1	429	0.0	826	G	bacteNOG00	G	bactNOG005	G	bctoNOG007	G
IL_F5_CDS_26	585543.HMPREF0969_00346	99.73	750	2	0	1	750	1	750	0.0	1489	G	bacteNOG04	G	bactNOG024	G	bctoNOG006	G
IL_F5_CDS_27	585543.HMPREF0969_00345	94.43	431	22	2	1	430	3	432	0.0	830	S	bactNOG198	S	bctoNOG056	S	NA	NA
IL_F5_CDS_28	585543.HMPREF0969_00344	88.93	289	32	0	26	314	165	453	0.0	553	G	bactNOG525	G	NA	NA	NA	NA
IL_B1_CDS_22	585543.HMPREF0969_00340	98.44	963	15	0	21	983	1	963	0.0	1847	S	bactNOG008	S	bctoNOG009	S	NA	NA
IL_B1_CDS_21	585543.HMPREF0969_00338	97.79	181	4	0	1	181	1	181	4,00E-130	368	S	bacteNOG03	S	bactNOG146	S	bctoNOG168	S
IL_B1_CDS_20	585543.HMPREF0969_00337	99.68	317	1	0	1	317	1	317	0.0	627	M	bacteNOG00	M	bactNOG014	M	bctoNOG004	M
IL_B1_CDS_19	585543.HMPREF0969_00336	98.76	161	2	0	1	161	1	161	1,00E-114	328	S	bacteNOG07	S	bactNOG170	S	bctoNOG183	S
IL_B1_CDS_18	585543.HMPREF0969_00335	99.22	255	2	0	6	260	1	255	5,00E-175	488	S	bacteNOG06	S	bactNOG113	S	bctoNOG129	S
IL_B1_CDS_17	585543.HMPREF0969_00334	100.00	192	0	0	1	192	1	192	3,00E-131	372	S	bacteNOG03	S	bactNOG710	S	bctoNOG139	S
IL_B1_CDS_16	585543.HMPREF0969_00333	99.11	338	3	0	1	338	1	338	0.0	700	H	bacteNOG01	H	bactNOG013	H	bctoNOG057	H
IL_B1_CDS_15	585543.HMPREF0969_00332	100.00	122	0	0	1	122	1	122	1,00E-71	216	S	bacteNOG02	S	bactNOG942	S	bctoNOG124	S
IL_B1_CDS_14	585543.HMPREF0969_00331	98.80	249	3	0	1	249	1	249	5,00E-175	488	D	bacteNOG01	D	bactNOG007	D	bctoNOG066	D
IL_B1_CDS_13	585543.HMPREF0969_00330	99.16	476	4	0	1	476	1	476	0.0	979	S	bacteNOG00	S	bactNOG169	S	bctoNOG013	S
IL_B1_CDS_12	585543.HMPREF0969_00329	100.00	156	0	0	1	156	1	156	1,00E-105	304	S	bacteNOG03	S	bactNOG120	S	bctoNOG143	S
IL_B1_CDS_11	585543.HMPREF0969_00328	100.00	333	0	0	1	333	1	333	0.0	612	S	bactNOG038	S	NA	NA	NA	NA
IL_B1_CDS_10	585543.HMPREF0969_00327	98.97	292	3	0	1	292	1	292	0.0	602	F	bacteNOG00	F	bctoNOG004	F	NA	NA
IL_B1_CDS_9	585543.HMPREF0969_00326	98.92	464	5	0	1	464	1	464	0.0	939	R	bacteNOG00	R	bactNOG001	R	bctoNOG001	R
IL_B1_CDS_7	585543.HMPREF0969_00195	99.72	359	1	0	1	359	1	359	0.0	659	G	bacteNOG01	G	bactNOG075	G	bctoNOG009	G
IL_B1_CDS_6	763034.HMPREF9446_00851	89.54	478	50	0	1	478	59	536	0.0	825	C	bacteNOG01	C	bctoNOG013	C	NA	NA
IL_B1_CDS_5	585543.HMPREF0969_00193	100.00	196	0	0	1	196	1	196	4,00E-143	402	S	bacteNOG02	S	bactNOG965	S	bctoNOG117	S
IL_B1_CDS_4	585543.HMPREF0969_00192	100.00	220	0	0	1	220	1	220	2,00E-151	425	K	bacteNOG02	K	bactNOG769	K	bctoNOG096	K
IL_B1_CDS_3	585543.HMPREF0969_00191	99.44	354	2	0	1	354	13	366	0.0	674	J	bacteNOG00	J	bactNOG008	J	bctoNOG004	J
IL_B1_CDS_2	585543.HMPREF0969_00190	99.77	428	1	0	1	428	1	428	0.0	828	E	bacteNOG01	E	bactNOG001	E	bctoNOG010	E
IL_B1_CDS_1	585543.HMPREF0969_00189	98.33	60	1	0	34	93	2	61	4,00E-38	138	S	bacteNOG03	S	bactNOG198	S	bctoNOG002	S

Table S1 (continued):

CDS	Hit name[a]	identity (%)	alignment le	mismatches	gap opening	q. start	q. end	s. start	s. end	e-value	bit score	EggNOGG category[b]	1st assignati	1st letter	2nd assignati	2nd letter	3rd assignati	3rd letter
IL_B5_CDS_2	657318.EUR_02280	97.69	390	5	1	1	386	1	390	0.0	765	G	bactNOG228	G	firmNOG056	G	NA	NA
IL_B5_CDS_3	657318.EUR_02270	99.09	882	8	0	1	882	1	882	0.0	1736	P	bactNOG003	P	firmNOG000	P	NA	NA
IL_B5_CDS_4	657318.EUR_02260	100.00	106	0	0	20	125	20	125	7,00E-72	216	S	bactNOG252	S	NA	NA	NA	NA
IL_B5_CDS_5	657318.EUR_02250	98.06	103	2	0	1	103	1	103	4,00E-68	205	S	bactNOG247	S	firmNOG268	S	NA	NA
IL_B5_CDS_6	657318.EUR_01980	98.24	397	7	0	1	397	1	397	0.0	776	E	bactNOG228	E	firmNOG001	E	NA	NA
IL_B5_CDS_7	657318.EUR_01970	96.24	186	7	0	44	229	744	929	1,00E-122	376	T	bactNOG028	T	firmNOG003	T	NA	NA
IL_B5_CDS_8	657319.EUS_08260	100.00	76	0	0	1	76	1	76	4,00E-47	150	L	bactNOG143	L	firmNOG276	L	NA	NA
IL_B5_CDS_9	657319.EUS_08270	100.00	125	0	0	1	125	12	136	1,00E-70	214	S	bactNOG104	S	firmNOG182	S	NA	NA
IL_B5_CDS_10	717960.EC1_00970	100.00	101	0	0	1	101	1	101	3,00E-67	203	S	bactNOG108	S	firmNOG182	S	NA	NA
IL_B5_CDS_11	657313.RTO_31030	97.56	573	14	0	1	573	1	573	0.0	1121	L	bactNOG093	L	firmNOG037	L	NA	NA
IL_B5_CDS_12	717962.CC1_31590	100.00	236	0	0	14	249	1	236	1,00E-154	436	S	bactNOG345	S	firmNOG042	S	NA	NA
IL_B5_CDS_13	411461.DORFOR_02646	99.26	270	2	0	1	270	1	270	3,00E-164	462	T	bactNOG067	T	firmNOG108	T	NA	NA
IL_B5_CDS_14	657318.EUR_03800	100.00	310	0	0	1	310	1	310	0.0	631	T	bactNOG000	T	firmNOG079	T	NA	NA
IL_B5_CDS_15	657318.EUR_29160	98.93	559	6	0	1	559	1	559	0.0	1139	L	bactNOG230	L	firmNOG472	L	NA	NA
IL_B5_CDS_21	657318.EUR_29220	100.00	220	0	0	1	220	1	220	1,00E-160	449	L	firmNOG361	L	NA	NA	NA	NA
IL_B5_CDS_22	657318.EUR_29230	98.83	426	5	0	1	426	1	426	0.0	845	L	bactNOG175	L	firmNOG339	L	NA	NA
IL_B5_CDS_24	657318.EUR_29250	99.35	155	1	0	1	155	1	155	4,00E-92	270	S	bactNOG956	S	firmNOG182	S	NA	NA
IL_B5_CDS_28	657318.EUR_29810	85.37	82	8	2	94	172	134	214	1,00E-40	142	F	bactNOG230	F	NA	NA	NA	NA
IL_B5_CDS_30	657318.EUR_29830	97.80	182	4	0	1	182	1	182	2,00E-114	329	S	bactNOG171	S	firmNOG313	S	NA	NA
IL_B5_CDS_34	657318.EUR_29870	96.15	519	20	0	1	519	1	519	0.0	988	R	bactNOG750	R	firmNOG198	R	NA	NA
IL_B3_CDS_2	537007.BLAHAN_04440	83.78	450	73	0	1	450	1	450	0.0	734	S	bactNOG207	S	firmNOG049	S	NA	NA
IL_B3_CDS_3	537007.BLAHAN_04443	86.26	262	36	0	16	277	15	276	9,00E-161	454	G	bactNOG045	G	firmNOG114	G	NA	NA
IL_B3_CDS_4	537007.BLAHAN_04444	89.12	294	32	0	1	294	1	294	0.0	540	G	bactNOG107	G	firmNOG050	G	NA	NA
IL_B3_CDS_5	457421.CBFG_01817	83.90	410	64	1	1	408	23	432	0.0	693	G	bactNOG228	G	firmNOG038	G	NA	NA
IL_B3_CDS_6	537007.BLAHAN_04446	82.82	326	56	0	1	326	1	326	0.0	545	K	firmNOG021	K	NA	NA	NA	NA
IL_B3_CDS_7	658655.HMPREF0988_01971	94.83	116	6	0	1	116	504	619	2,00E-70	228	V	bactNOG000	V	firmNOG005	V	NA	NA
IL_B3_CDS_10	657313.RTO_31960	95.38	65	3	0	5	69	6	70	2,00E-39	135	S	bactNOG223	S	firmNOG033	L	NA	NA
IL_B3_CDS_11	657313.RTO_31950	100.00	63	0	0	1	63	1	63	1,00E-36	123	S	bactNOG161	S	firmNOG310	S	NA	NA
IL_B3_CDS_12	657318.EUR_02310	99.62	524	2	0	1	524	1	524	0.0	1038	F	bactNOG014	F	firmNOG003	F	NA	NA
IL_B3_CDS_13	657318.EUR_02300	97.44	195	5	0	1	195	1	195	7,00E-122	349	S	bactNOG109	S	firmNOG038	S	NA	NA
IL_B3_CDS_15	657318.EUR_02280	97.69	390	5	1	1	386	1	390	0.0	762	G	bactNOG228	G	firmNOG056	G	NA	NA
IL_B3_CDS_16	657318.EUR_02270	99.32	882	6	0	1	882	1	882	0.0	1741	P	bactNOG003	P	firmNOG000	P	NA	NA
IL_B3_CDS_17	657318.EUR_02260	99.06	106	1	0	20	125	20	125	4,00E-71	214	S	bactNOG252	S	NA	NA	NA	NA
IL_B3_CDS_18	657318.EUR_02250	98.06	103	2	0	1	103	1	103	4,00E-68	205	S	bactNOG247	S	firmNOG268	S	NA	NA
IL_B3_CDS_19	657318.EUR_02240	99.57	1388	6	0	1	1388	1	1388	0.0	2810	D	bactNOG063	D	firmNOG000	D	NA	NA
IL_H1_CDS_22	657318.EUR_02240	98.26	172	3	0	1	172	760	931	9,00E-109	345	D	bactNOG063	D	firmNOG000	D	NA	NA
IL_H1_CDS_23	657318.EUR_02240	99.37	319	2	0	1	319	1070	1388	0.0	655	D	bactNOG063	D	firmNOG000	D	NA	NA
IL_H1_CDS_24	657318.EUR_02250	98.06	103	2	0	1	103	1	103	4,00E-68	205	S	bactNOG247	S	firmNOG268	S	NA	NA
IL_H1_CDS_25	657318.EUR_02260	99.06	106	1	0	20	125	20	125	4,00E-71	214	S	bactNOG252	S	NA	NA	NA	NA
IL_H1_CDS_26	657318.EUR_02270	99.32	882	6	0	1	882	1	882	0.0	1741	P	bactNOG003	P	firmNOG000	P	NA	NA
IL_H1_CDS_27	657318.EUR_02280	97.69	390	5	1	1	386	1	390	0.0	762	G	bactNOG228	G	firmNOG056	G	NA	NA
IL_H1_CDS_29	657318.EUR_02300	96.41	195	7	0	1	195	1	195	4,00E-120	344	S	bactNOG109	S	firmNOG038	S	NA	NA
IL_H1_CDS_30	657318.EUR_02310	99.62	524	2	0	1	524	1	524	0.0	1038	F	bactNOG014	F	firmNOG003	F	NA	NA
IL_H1_CDS_31	657313.RTO_31950	100.00	63	0	0	1	63	1	63	1,00E-36	123	S	bactNOG161	S	firmNOG310	S	NA	NA
IL_H1_CDS_32	657313.RTO_31960	95.38	65	3	0	5	69	6	70	2,00E-39	135	S	bactNOG223	S	firmNOG033	L	NA	NA
IL_H1_CDS_35	658655.HMPREF0988_01971	94.83	116	6	0	1	116	504	619	2,00E-70	228	V	bactNOG000	V	firmNOG005	V	NA	NA
IL_H1_CDS_36	537007.BLAHAN_04446	82.82	326	56	0	1	326	1	326	0.0	545	K	firmNOG021	K	NA	NA	NA	NA
IL_H1_CDS_37	457421.CBFG_01817	83.90	410	64	1	1	408	23	432	0.0	693	G	bactNOG228	G	firmNOG038	G	NA	NA
IL_H1_CDS_38	537007.BLAHAN_04444	89.12	294	32	0	1	294	1	294	0.0	540	G	bactNOG107	G	firmNOG050	G	NA	NA
IL_H1_CDS_39	537007.BLAHAN_04443	86.26	262	36	0	16	277	15	276	9,00E-161	454	G	bactNOG045	G	firmNOG114	G	NA	NA

[a] Best hits from BlastP homology to proteins from the eggNOG 4.0 database (61) (E-value ≤ 10-7, identity percentage ≥ 80%).

[b] according to Powell, S. et al. eggNOG v4.0: nested orthology inference across 3686 organisms. Nucleic acids research 42, D231-239, doi:10.1093/nar/gkt1253 (2014).

Table S2: see the separate Excel file.

Table S3: Putative activities of the evidenced Glycoside Hydrolase (GH) and Carbohydrate Esterase (CE) families in the eleven positive clones.

**GH2**	β-galactosidase (EC 3.2.1.23) ; β-mannosidase (EC 3.2.1.25); β-glucuronidase (EC 3.2.1.31); α-L-arabinofuranosidase (EC 3.2.1.55); mannosylglycoprotein endo-β-mannosidase (EC 3.2.1.152); exo-β-glucosaminidase (EC 3.2.1.165)
**GH3**	β-glucosidase (EC 3.2.1.21); xylan 1,4-β-xylosidase (EC 3.2.1.37); β-glucosylceramidase (EC 3.2.1.45); β-N-acetylhexosaminidase (EC 3.2.1.52); α-L-arabinofuranosidase (EC 3.2.1.55); glucan 1,3-β-glucosidase (EC 3.2.1.58); glucan 1,4-β-glucosidase (EC 3.2.1.74); isoprimeverose-producing oligoxyloglucan hydrolase (EC 3.2.1.120); coniferin β-glucosidase (EC 3.2.1.126); exo-1,3-1,4-glucanase (EC 3.2.1.-); β-N-acetylglucosaminide phosphorylases (EC 2.4.1.-)
**GH5**	endo-β-1,4-glucanase / cellulase (EC 3.2.1.4); endo-β-1,4-xylanase (EC 3.2.1.8); β-glucosidase (EC 3.2.1.21); β-mannosidase (EC 3.2.1.25); β-glucosylceramidase (EC 3.2.1.45); glucan β-1,3-glucosidase (EC 3.2.1.58); licheninase (EC 3.2.1.73); exo-β-1,4-glucanase / cellodextrinase (EC 3.2.1.74); glucan endo-1,6-β-glucosidase (EC 3.2.1.75); mannan endo-β-1,4-mannosidase (EC 3.2.1.78); cellulose β-1,4-cellobiosidase (EC 3.2.1.91); steryl β-glucosidase (EC 3.2.1.104); endoglycoceramidase (EC 3.2.1.123); chitosanase (EC 3.2.1.132); β-primeverosidase (EC 3.2.1.149); xyloglucan-specific endo-β-1,4-glucanase (EC 3.2.1.151); endo-β-1,6-galactanase (EC 3.2.1.164); hesperidin 6-O-α-L-rhamnosyl-β-glucosidase (EC 3.2.1.168); β-1,3-mannanase (EC 3.2.1.-); arabinoxylan-specific endo-β-1,4-xylanase (EC 3.2.1.-); mannan transglycosylase (EC 2.4.1.-)
GH5 subfamily 2	endo-β-1,4-glucanase (EC 3.2.1.4); endo-β-1,4-xylanase (EC 3.2.1.8); chitosane (EC 3,2,1,132)
GH5 subfamily 7	endo β-1,4-mannosidase (EC 3.2.1.78); mannan transglycosylase (EC 2.4.1.-); β-mannosidase (EC 3.2.1.25)
**GH9**	endoglucanase (EC 3.2.1.4); endo-β-1,3(4)-glucanase / lichenase-laminarinase (EC 3.2.1.6); β-glucosidase (EC 3.2.1.21); lichenase / endo-β-1,3-1,4-glucanase (EC 3.2.1.73); exo-β-1,4-glucanase / cellodextrinase (EC 3.2.1.74); cellobiohydrolase (EC 3.2.1.91); xyloglucan-specific endo-β-1,4-glucanase / endo-xyloglucanase (EC 3.2.1.151); exo-β-glucosaminidase (EC 3.2.1.165)
**GH13**	α-amylase (EC 3.2.1.1); pullulanase (EC 3.2.1.41); cyclomaltodextrin glucanotransferase (EC 2.4.1.19); cyclomaltodextrinase (EC 3.2.1.54); trehalose-6-phosphate hydrolase (EC 3.2.1.93); oligo-α-glucosidase (EC 3.2.1.10); maltogenic amylase (EC 3.2.1.133); neopullulanase (EC 3.2.1.135); α-glucosidase (EC 3.2.1.20); maltotetraose-forming α-amylase (EC 3.2.1.60); isoamylase (EC 3.2.1.68); glucodextranase (EC 3.2.1.70); maltohexaose-forming α-amylase (EC 3.2.1.98); maltotriose-forming α-amylase (EC 3.2.1.116); branching enzyme (EC 2.4.1.18); trehalose synthase (EC 5.4.99.16); 4-α-glucanotransferase (EC 2.4.1.25); maltopentaose-forming α-amylase (EC 3.2.1.-) ; amylosucrase (EC 2.4.1.4) ; sucrose phosphorylase (EC 2.4.1.7); malto-oligosyltrehalose trehalohydrolase (EC 3.2.1.141); isomaltulose synthase (EC 5.4.99.11); malto-oligosyltrehalose synthase (EC 5.4.99.15); amylo-α-1,6-glucosidase (EC 3.2.1.33); α-1,4-glucan: phosphate α-maltosyltransferase (EC 2.4.99.16); 6?-P-sucrose phosphorylase (EC 2.4.1.-); amino acid transporter.
**GH16**	xyloglucan:xyloglucosyltransferase (EC 2.4.1.207); keratan-sulfate endo-1,4-β-galactosidase (EC 3.2.1.103); endo-1,3-β-glucanase (EC 3.2.1.39); endo-1,3(4)-β-glucanase (EC 3.2.1.6); licheninase (EC 3.2.1.73); β-agarase (EC 3.2.1.81); κ-carrageenase (EC 3.2.1.83); xyloglucanase (EC 3.2.1.151); endo-β-1,3-galactanase (EC 3.2.1.181); β-porphyranase (EC 3.2.1.178); hyaluronidase (EC 3.2.1.35); endo-β-1,4-galactosidase (EC 3.2.1.-); chitin β-1,6-glucanosyltransferase (EC 2.4.1.-); endo-β-1,4-galactosidase (EC 3.2.1.-)
**GH20**	β-hexosaminidase (EC 3.2.1.52); lacto-N-biosidase (EC 3.2.1.140); β-1,6-N-acetylglucosaminidase) (EC 3.2.1.-); β-6-SO3-N-acetylglucosaminidase (EC 3.2.1.-)
**GH31**	α-glucosidase (EC 3.2.1.20); α-galactosidase (EC 3.2.1.22); α-mannosidase (EC 3.2.1.24); α-1,3-glucosidase (EC 3.2.1.84); sucrase-isomaltase (EC 3.2.1.48) (EC 3.2.1.10); α-xylosidase (EC 3.2.1.177); α-glucan lyase (EC 4.2.2.13); isomaltosyltransferase (EC 2.4.1.-); oligosaccharide α-1,4-glucosyltransferase (EC 2.4.1.161)
**GH32**	invertase (EC 3.2.1.26); endo-inulinase (EC 3.2.1.7); β-2,6-fructan 6-levanbiohydrolase (EC 3.2.1.64); endo-levanase (EC 3.2.1.65); exo-inulinase (EC 3.2.1.80); fructan β-(2,1)-fructosidase/1-exohydrolase (EC 3.2.1.153); fructan β-(2,6)-fructosidase/6-exohydrolase (EC 3.2.1.154); sucrose:sucrose 1-fructosyltransferase (EC 2.4.1.99); fructan:fructan 1-fructosyltransferase (EC 2.4.1.100); sucrose:fructan 6-fructosyltransferase (EC 2.4.1.10); fructan:fructan 6G-fructosyltransferase (EC 2.4.1.243); levan fructosyltransferase (EC 2.4.1.-); [retaining] sucrose:sucrose 6-fructosyltransferase (6-SST) (EC 2.4.1.-); cycloinulo-oligosaccharide fructanotransferase (EC 2.4.1.-)
**GH91**	inulin lyase [DFA-I-forming] (EC 4.2.2.17); inulin lyase [DFA-III-forming] (EC 4.2.2.18); difructofuranose 1,2':2,3' dianhydride hydrolase [DFA-IIIase] (EC 3.2.1.-)
**GH94**	cellobiose phosphorylase (EC 2.4.1.20); laminaribiose phosphorylase (EC 2.4.1.31); cellodextrin phosphorylase (EC 2.4.1.49); chitobiose phosphorylase (EC 2.4.1.-); cyclic β-1,2-glucan synthase (EC 2.4.1.-); cellobionic acid phosphorylase (EC 2.4.1.321); β-1,2-oligoglucan phosphorylase (EC 2.4.1.-)
**GH97**	glucoamylase (EC 3.2.1.3); α-glucosidase (EC 3.2.1.20); α-galactosidase (EC 3.2.1.22)
**GH127**	β-L-arabinofuranosidase (EC 3.2.1.185)
**CE4**	acetyl xylan esterase (EC 3.1.1.72); chitin deacetylase (EC 3.5.1.41); chitooligosaccharide deacetylase (EC 3.5.1.-); peptidoglycan GlcNAc deacetylase (EC 3.5.1.-); peptidoglycan N-acetylmuramic acid deacetylase (EC 3.5.1.-).
**CE7**	acetyl xylan esterase (EC 3.1.1.72); cephalosporin-C deacetylase (EC 3.1.1.41).

[a]: according to the CAZY website (www.cazy.org), update from april 2016.

Legend:

- exo-hydrolase containing family
- endo-hydrolase containing family
- exo and endo-hydrolase containing family

Table S4: see the separate Excel file.

Table S5: see the separate Excel file.
